# Cre-Lox miRNA-delivery technology optimized for inducible microRNA and gene-silencing studies in zebrafish

**DOI:** 10.1093/nar/gkaf004

**Published:** 2025-01-20

**Authors:** Fangfei Guo, Alisha Tromp, Haitao Wang, Thomas E Hall, Jean Giacomotto

**Affiliations:** Institute for Biomedicine and Glycomics, School of Environment and Science, Griffith University, 46 Don Young Road, Brisbane QLD 4111, Australia., Brisbane, QLD 4111, Australia; Queensland Brain Institute, The University of Queensland, Building 79 Research Lane, Brisbane, Queensland 4072, Australia; Queensland Brain Institute, The University of Queensland, Building 79 Research Lane, Brisbane, Queensland 4072, Australia; Institute for Molecular Bioscience, The University of Queensland, 306 Carmody Road, Brisbane, Queensland 4072, Australia; Institute for Biomedicine and Glycomics, School of Environment and Science, Griffith University, 46 Don Young Road, Brisbane QLD 4111, Australia., Brisbane, QLD 4111, Australia; Queensland Brain Institute, The University of Queensland, Building 79 Research Lane, Brisbane, Queensland 4072, Australia; Thomson Institute, National PTSD Research Centre, University of the Sunshine Coast, 12 Innovation Parkway, Birtinya, Queensland 4575, Australia

## Abstract

While many genetic tools exist for zebrafish, this animal model still lacks robust gene-silencing and microRNA-delivery technologies enabling spatio-temporal control and traceability. We have recently demonstrated that engineered *pri-miR* backbones can trigger stable gene knockdown and/or express microRNA(s) of choice in this organism. However, this miRNA-expressing technology presents important limitations. First, to trigger potent knockdown(s), multiple synthetic-miRNAs must be expressed simultaneously, compromising the co-expression of fluorescent marker(s) and knockdown traceability. Second, when gene(s) knockdown triggers significant phenotypes, like homozygous mutants with severe early phenotypes, it is difficult, if not impossible, to maintain transgenic carriers. To solve these problems and provide a mature RNAi and microRNA-delivery technology, we have generated new RNAi reagents and an inducible delivery system based on the Cre/Lox technology. This system allows the creation of asymptomatic/silent carriers, easing the production of embryos with potent knockdowns that can be traced and spatiotemporally controlled. We further demonstrated the utility of this approach by establishing novel inducible and tissue-specific models of spinal muscular atrophy, opening new avenues for studying *smn1*-gene function and pathogenicity. All in all, these materials and techniques will be invaluable in studying microRNA biology and in modelling or tackling conditions in which gene dosage is key.

## Graphical Abstract

**Graphical Abstract:**
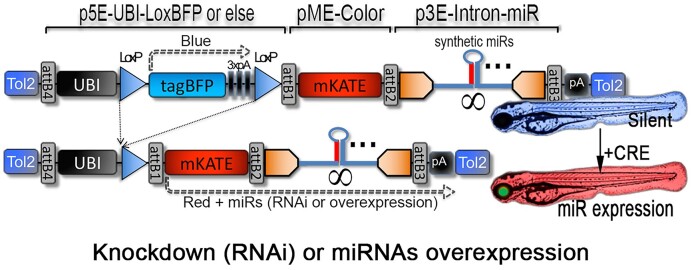

## Introduction

The zebrafish has become a powerful animal model for investigating gene function, modelling human diseases and screening for bioactive compounds. It combines the tractability and versatility of *Caenorhabditis elegans* and *Drosophila melanogaster* with the ability to replicate/model human disorders and enable screens for drug discovery and chemical genetics. Nonetheless, one key technology that has been missing and remained intractable is a robust methodology for RNA interference (RNAi, gene-silencing or knockdown) and microRNA-delivery (1–4). To develop such tools for zebrafish, we have recently tested a variety of approaches and found that an engineered *pri-miR* (primary-miRNA) can be used to release any *pre-miR* (precursor-miRNA) and mature miRNA(s) of choice for either microRNA basic research studies and/or to silence endogenous gene(s)-of-interest throughout the entire lifespan of the fish (5–7). In practice, and as presented previously (5), a RNAi cassette containing (i) a polymerase-II promoter, (ii) a fluorescent marker and (iii) a custom *pri-miR*(s) [designed to release a synthetic microRNA (miR) against the 3′UTR of a gene-of-interest] is cloned into a Tol2-transgene for genomic integration (8). Upon activation, this transgene triggers co-expression of a fluorescent marker and the synthetic-miR(s), thereby silencing the targeted gene(s) while highlighting/marking the affected cells (Supplementary Figure S1A).

**Figure 1.**
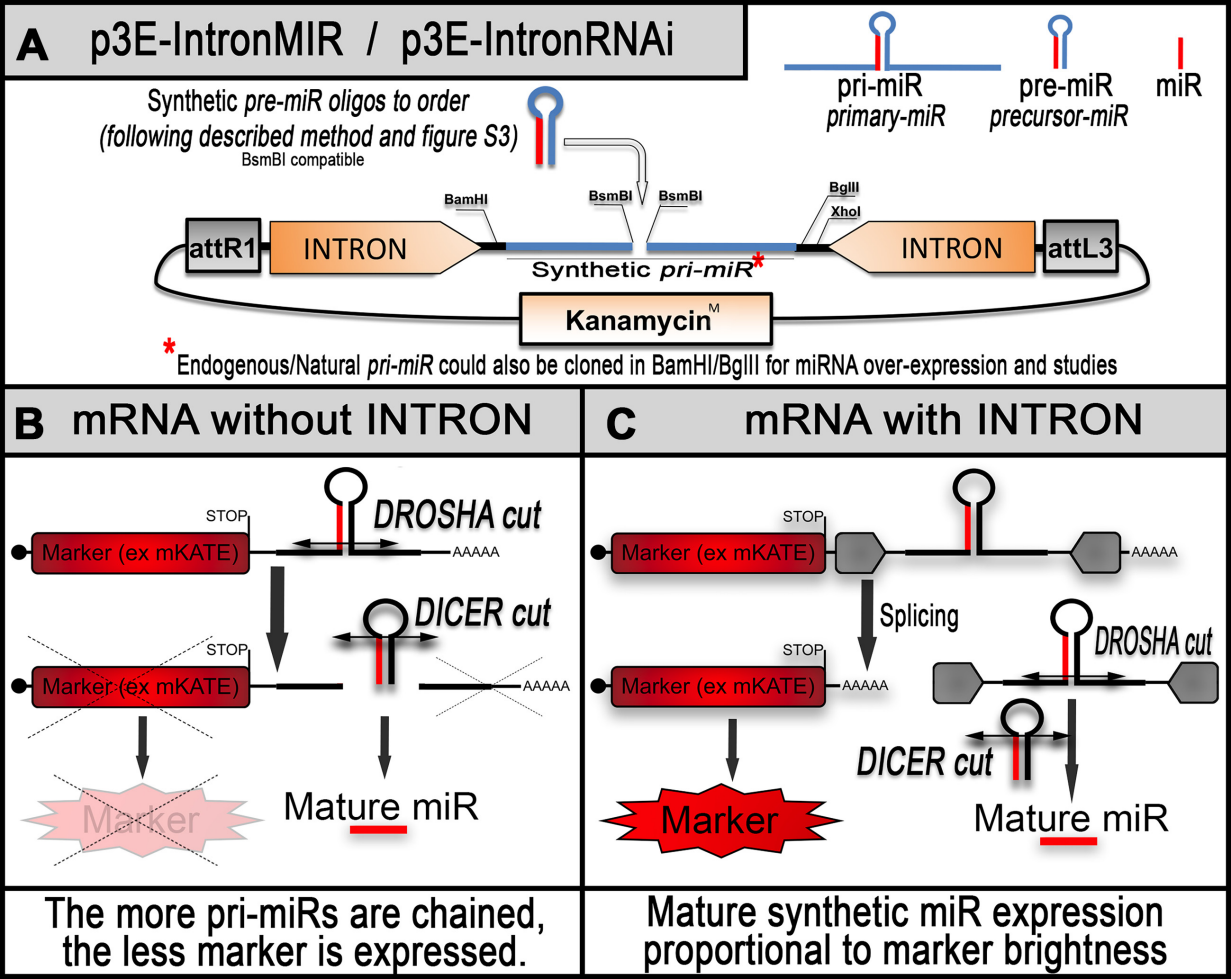
Schematics of the new RNAi backbone for zebrafish gene silencing. (**A**) Tol2kit compatible p3E presenting an optimized empty synthetic *pri-miR* (or RNAi cassette) embedded into a β-globin intronic sequence. This presented *pri-miR* is designed to allow rapid directional insertion of synthetic *pre-miR* of choice. Synthetic p*re-miR(s)* of choice are generated by annealing specific top and bottom RNAi oligos designed to release a mature miRNA directed against the 3′UTR of a gene-of-interest [see ‘Methods’ section and previous material (5)]. The RNAi cassette is flanked by restriction sites allowing subsequent and repetitive chaining [see ‘Methods’ and Supplementary Figure S2) for generating p3E-RNAi plasmid with multiple *pri-miR* for either increasing the potency of the desired knockdown or targeting multiple genes at the same time. (**B**) Without intron, the *pre-miR*/RNAi cassette is transcribed along with a co-marker on the same RNA, which is cut by Drosha in the nucleus for releasing the associated *pre-miR*. This cut leaves the mRNA without polyA tail and leads to its rapid degradation. The associated fluorescence is not a good indicator of the activity and amount of synthetic miRNA produced. (**C**) The presence of the intronic sequence is designed to rescue the co-expression of the marker (see also Supplementary Figure S1).

Although this technology opens new avenues of research for zebrafish genetics, unfortunately, to date, this RNAi methodology still presents important limitations to apply to any gene-of-interest and to multi-genic knockdown. First, to trigger potent knockdown, one must express multiple synthetic miRNAs against different target sites located on the gene-of-interest’s 3′UTR. To achieve this, multiple *pri-miRs* must be chained as concatemer (Supplementary Figure S2A). However, after >3× chained *pri-miRs*, we found it difficult to generate transgenic lines with visible co-expression of fluorescence [see (7) and below as an example for lines targeting the gene *smn1*]. This loss of fluorescence is likely due to the requirement for the *pri-miRs* to first be processed/cut via Drosha and Dicer to generate mature miRs. These successive cuts leave the associated mRNA without polyA tails thereby triggering their degradation (Supplementary Figure S1A and Figure 1B). In this model, the miRs processing/maturation directly competes with the translation of the attached fluorescent marker, with the more *pri-miRs*/miRNAs chained, the less fluorescent protein successfully produced. Second, when the gene knockdown triggers significant phenotypes, similar to homozygous mutants with strong early defects, it becomes challenging to maintain stable transgenic lines with even one copy of the RNAi-transgene. This can be a significant problem when one tries to model human diseases such as spinal muscular atrophy (SMA) (5,6,9) which are associated with SMN haploinsufficiency/partial loss-of-function (LOF). Expression of miRNAs against the causal gene *smn1* triggers strong early motor neuron defects, motor function loss and premature death consistent with disease presentation. However, the more severe the phenotype, the more difficult it becomes to both isolate and maintain animals.

To solve these problems and deliver a mature RNAi and microRNA-delivery technology for zebrafish, we have generated and validated (i) new reagents that are compatible with the zebrafish Tol2kit (10) and that offer the possibility to chain numerous synthetic or natural *pre-miR* without affecting the co-expression of a fluorescent marker as well as (ii) an inducible system based on Cre/Lox technology allowing the generation of conditional lines that can be induced with spatiotemporal control. Subsequently, using this system, we generated a versatile conditional zebrafish model of SMA. This model is opening new avenues for investigating Smn function and for large-scale experiments such as drug screenings.

Finally, this system promises to be very useful to the community to express and study endogenous miRNA(s).

## Materials and methods

### Zebrafish maintenance

Adult zebrafish and embryos were maintained by standard protocols approved by the University of Queensland and Griffith University Animal Ethics Committee. Ethics approval AE213_18/AE213_18 and GRIDD/11/22/AEC. Wild-type lines used in these studies are Tubingen/AB (TAB) background.

### Cloning of p3E-RNAi

We first cloned a p3E clone incorporating the rabbit beta-globin intron presented here (‘Kaloop’ plasmid) (11). The intronic sequence along with a SV40 signal was amplified using forward, Rabbit_GI_F1_p3E (ggggacagctttcttgtacaaagtggCTCGACCGATCCTGAGAACTT) and reverse primer, SV40_late_pA_R1_p3E (ggggacaactttgtataataaagttgCCACACCTCCCCCTGAAC). The generated PCR (polymerase chain reaction) product was subsequently recombined into pDONRp2R-p3 using Gateway LR clones following manufacturer instructions. The final plasmid was named 303_p3E_GI_PA. We then introduced into this 303_p3E_GI_PA our previous RNAi cassette from plasmid pME-RNAi641 (5) using an ‘In-fusion (Takara)’ cloning strategy. We digested 303_p3E_GI_PA using DraI (middle portion of the beta-globin intronic sequence) and purified it. Concomitantly, we amplified the previously published RNAi cassette using In-fusion compatible forward (TTGTAACGAATTTTTcgtcgatcgtttaaagggagg) and reverse-primer (TTGTAACGAATTTTTcgtcgatcgtttaaagggagg). We conducted an In-fusion cloning reaction (In Fusion HD kit, Takara) following the manufacturer’s instructions to introduce the RNAi cassette into the digested intronic sequence. The kanamycin gene resistance sequence in the resulting clone presented an XhoI site that would interfere with the chaining ability of the RNAi cassette. We then removed it using a site directed mutagenesis using Phusion High Fidelity DNA Polymerase and the following forward and reverse primers (CGATCGCGTATTTCGcCTCGCTCAGGCGCAA and TTGCGCCTGAGCGAGgCGAAATACGCGATCG). The reaction conducted was as follows, first denaturation step at 98°C (15 s) followed by 18 cycles of 98°C (15 s)/65°C (30 s)/72°C (1 min) and terminated by an elongation step 72°C (5 min). A sample of 5 μl was run on a gel to verify the presence of a single appropriately sized band. The remaining reaction mix was then treated with 0.3 μl of DpnI for 10 min at 37°C. A volume of 2 μl was finally used to transformed competent cells and plated on plates supplement with kanamycin. The resulting colonies were screened by miniprep and sequencing. The final plasmid was named p3E-IntronRNAi (addgene #163380).

### Artificial/synthetic *pri-miRNA* design, assembly and insertion into p3E-IntronRNAi

In order to design a *pre-miR* to insert into the p3E-IntronMIR, the minimal 3′UTR region of the gene(s) of interest should first be identified and confirmed. Targetscan Fish (http://www.targetscan.org/) provide useful resources in addition to traditional online databases such as *Ensembl*. However, we strongly recommend sequencing the 3′UTR of interest to confirm the integrity of the identified sequence on the mRNA(s) to deal with any potential polymorphisms or isoform specificity. Once confirmed, target locations should be identified manually or using online tools. We recommend the miRNA-design tool provided by Invitrogen (www.genscript.com/design_center.html), which generates oligos compatible with p3E-RNAi, thereby ready for order. For a manual design, follow the guidelines in Supplementary Figure S3. Other possible online tools are described in the original method (5). After the design, order the top and bottom *pre-miRNA* oligos using your favourite oligo provider, and resuspend them at 200 mM for storage. For generating the *pre-miR* to insert into BsmBI-digested p3E-IntronRNAi, anneal 5 μl of these 200 mM top and bottom RNAi oligonucleotides in a 20 μl reaction including 2 μl of NEB2 10× buffer. The mixture is heated at 95°C for 5 min in a thermocycler and left in the machine to cool down for another 30 min. Samples are then vortexed and briefly spun down before being diluted 5000-fold in water at room temperature. Diluted final *pre-miRNA* samples are stocked at room temperature until ligation into BsmBI-digested p3E-IntronRNAi. Discard after use. For inserting those *pre-miRNA*, p3E-IntronRNAi is first digested by BsmBI and gel extracted. The resulting linearised plasmid and pre-RNA are ligated as follows: Mix 10 ng of linearized p3E-IntronRNAi with 4 μl of the 5000-diluted *pre-miR* and ligated for 1 h at room temperature before transforming Max efficiency bacteria and plate on kanamycin. The next day screen your colonies with forward (46_F_RNAi aagggaggtagtgagtcgac) and reverse (47_F_RNAi ctagatatctcgagtgcggc) primers. We also recommend using those primers for sequencing. Note that BsmBI-digested p3E-IntronRNAi can be stored for several years after gel extraction and purification. Following the insertion of the annealed *pre-miR*, you will have generated functional *pri-miR* that can be chained as presented in Supplementary Figure S2 or as described below.

### Chaining of RNAi cassettes

**Figure 2.**
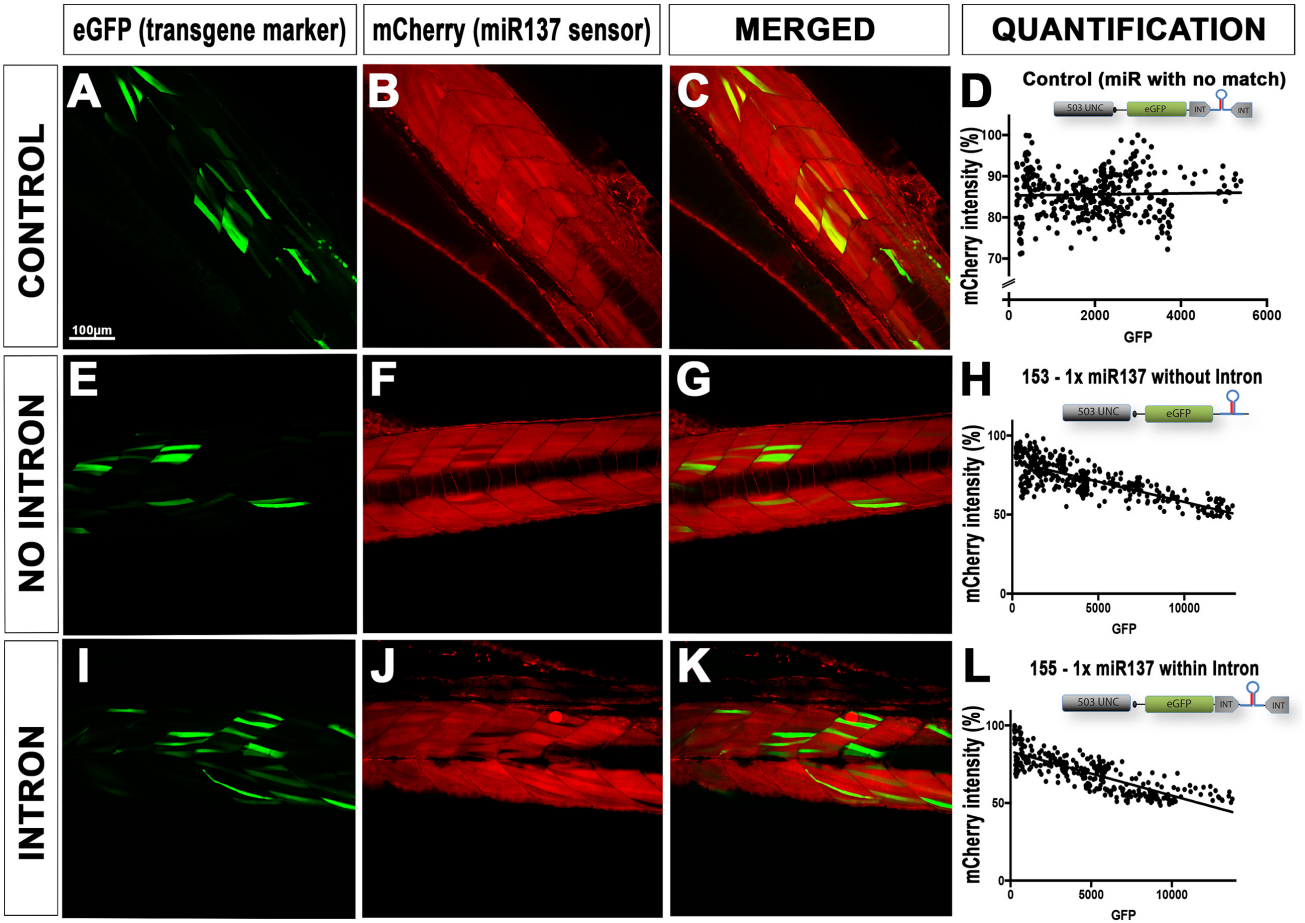
Validating effect of intron based RNAi approach on endogenous miRNA processing. (**A**–**C**), (**E**–**G**) and (**I**–**K**) Ubiquitously expressing mCherry fluorescent embryos from Tg(ubi:mCherry:SPmiR137) incrosses were injected with 25 pg of Tol2 transposase combined with 50 pg of control transgenes 157-control or anti-miR137 expressing constructs—153 (without intron), 155 (with intron). All injected plasmids led to mosaic eGFP expression. Plasmids 153 and 155, but not 157-control, led to a reduction of red fluorescence, hence knockdown of the integrated mCherry sensor transgene. A minimum of six larvae were analyzed per condition to estimate knockdown efficiency. Graphs D, H and L are representations of pixel intensities (each dot presents a grey value) of green versus red fluorescence (presented as a percentage) from z-stacks imaged with a confocal microscope at 4 day-post-fertilization (dpf). Scale bar is 100 μm.

Several *pre-miRNAs* used here, such as *pre-miR-137* and *pre-miRsmn1-4141*, have previously been cloned and described (5,12). For the 1× *miR137* constructs, previous *pri-miR-137* (12) have been gel extracted from UAS:YFPs:137 using BamHI/XhoI digestion, and inserted into p3E-IntronRNAi (also digested with BamHI/XhoI, gel extracted and purified), with the final plasmid named ‘p3E-Intron_miR137_1x’. *p3E-Intron_miR137-4x* (4× *pri-miR-137* repeats) was further generated through chaining of 4× *pri-miR-137* cassettes as presented in Supplementary Figure S2A. For generating *p3E-Intron-smn1-4141* used in this study, we gel-extracted the *pre-miRsmn1-4141* cassette from 641-dsRED-smn1-4141 (6), using BamHI/XhoI digestion. We further inserted this *anti-smn1* cassette into p3E-IntronRNAi for downstream cloning presented below. This p3E was named p3E-Intron-smn1-4141. Other chained pre-miRs used in this study followed the cloning protocol presented in Figure 2A.

### Transient experiments via RNAi-transgene injections

To validate the intron efficiency, we first generate Tol2-transgenes using a LR-reaction combining 394-pDestTol2pA2, 104_p5E-503unc (6), 383-pME-EGFP (Tol2kit) and the corresponding p3E with or without intron. We then injected 50 pg of each plasmid, named 153_503unc-eGFP_1xmiR137, 154_503unc-eGFP_4xmiR137, 155_503unc-eGFP_INT_1xmiR137, 156_503unc-eGFP_INT_4xmiR137 and controls along with 25 pg Tol2 transposase mRNA into 1-cell staged embryos (Supplementary Figure S4). These embryos were from an outcross of Tg(ubi:mCherry:SP137) fish ubiquitously expressing mCherry tagged with a custom 3′UTR recognised but *miR-137* (12). We imaged the animals (muscle fibres) at 4 dpf using confocal microscopy to ensure proper quantification of knockdown. We processed and analyzed the recordings using Fiji. To quantify pixel intensity, we used ‘plot profile’ to extract gray values across a line drawn across regions of interest. We plotted red fluorescence as a percentage and green fluorescence as raw values and generated linear regression curves using PRISM9.

### Generation of driver lines

Plasmid ubi:iCre, HuC:iCre and 503unc:iCre were produced using a 2-way Gateway LR reaction using manufacturer’s instructions. For all final plasmids, a custom R4-R2_destination clone named 1456-pDEST-minTol2_R4-R2_cryaa-eGFP (Addgene #171795, carrying a selection eGFP marker under the control of the Crystallin promoter) (13) was mixed with our 469-pME-iCre (#171792) (13) and either with 101_p5E-Ubiquitin, 102_p5E-HuC or 104_P5E-503unc. Each plasmid was injected into TAB fish at 25 ng.μl complexed with 25 ng.μl of transposase-mRNA for promoting DNA integration. Around 200 animals were injected and around 60 larvae were selected at 3 dpf based on visible green fluorescence expression in their eyes (lens). Larvae were raised until adulthood and screened for F0 founder/carrier. Three F0 adults were selected based on strong eGFP Lens-expression in their F1 progeny. The F1s were screened and raised until adulthood to be outcrossed with responder lines to test their ability to trigger Lox-recombination. Only one line was further maintained based on the validation/efficiency of the associated Cre activity.

### Cloning of p5E-ubi:LoxBFP

We first amplified tagBFP from our 15_pME_tagBFP stock plasmid using primers 101-BamHI-Flox-BFP-ForwRight (acagggatccaagcttataacttcgtatagcatacattatacgaagttatccggtcgccaccatgagcgagctgattaagg) and 102-BFP-NotI-Rev (AATTggatccgcggccgctttaattaagcttgtgccccagt). The primers were designed to insert a Flox sequence in 5′ of the tagBFP sequence, the overall amplicon being flanked by BamHI and NotI sites. The PCR product was further digested using BamHI along with Tol2kit 101_p5E-Ubiquitin plasmid (Addgene #27320) (14). Both were purified and 101_p5E-Ubiquitin was dephosphorylated using NEB Antarctic phosphatase following manufacturer’s instructions. T4 ligation was conducted overnight at 12°C and transformed using MAX Efficiency DH10B Competent Cells (Invitrogen #18297010). Bacteria were grown on kanamycin resistance plates overnight at 37°C. Ten colonies were amplified in 4 ml of LB supplemented with kanamycin. Plasmids were isolated through miniprep (PureLink Quick Plasmid Miniprep Kit) and sent for sequencing to confirm orientation of the insert and integration of the Lox_tagBFP cassette. The intermediate plasmid was named p5E-ubi_Lflox_BFP. We further digested it using NotI, purified and dephosphorylated it. In parallel, we gel extracted the 3× polyA sequence present on plasmid pENTR5'_ubi:loxP-EGFP-loxP (Addgene #27322) (15) and inserted it in the NotI-dephosphorylated p5E-ubi_Lflox_BFP plasmid through T4 ligation. Ten colonies were selected for miniprep as presented above and sequenced for selecting a clone with proper orientation. The final plasmid was named p5E-ubi-loxTagBFP (Addgene #172518).

### Cloning of ubi:loxBFP:dsRED:miRsmn1-4141 and ubi:loxBFP:mKate:INTRON_miRsmn1-4141

ubi:loxBFP:mKate:INTRON_miRsmn1-4141 was cloned using a Gateway LR-reaction mixing destination p5E-ubi-loxTagBFP (Addgene #172518) with our 010-pME-mKate2, p3E-Intron-smn1-4141 and destination clone 397-pDESTTol2pA_Cryaa-cherry. Reaction was transformed using Max efficiency bacteria and selected on ampicillin plate. PCR screening was performed, and two positive clones were sent for sequencing. ubi:loxBFP:dsRED:miRsmn1-4141 was generated through a similar cloning approach but mixing p5E-ubi-loxTagBFP with 641-dsRED-smn1-4141 and destination clone 1457-pDEST-minTol2_R4-R2_Cryaa-cherry.

### Generation of responder lines

Tol2-destination clones ubi:loxBFP:dsRED:miRsmn1-4141 and ubi:loxBFP:mKate:INTRON_miRsmn1-4141 were injected into one-cell stage tg(MN:GFP) embryos, with 30 ng.μl of corresponding DNA complexed with 25 ng.μl of transposase (5,16). Injected animals were screened at 3 dpf based on blue mosaic expression and/or red fluorescence expression in their eyes. Embryos were raised until adulthood and outcrossed with wild type (wt) to identify F0 founders that integrated the respective conditional RNAi transgenes, which was evidenced by both ubiquitous blue fluorescence expression and eye-specific red fluorescence emission. We selected the best six lines that we ranked based on their apparent blue fluorescence brightness and generated respective F1 stable lines. Once sexually mature, F1 lines were further outcrossed with tg(ubi:iCre) to characterise their ability to Flox/recombine and to express the conditional miR-cassettes, which was evaluated by observing and ranking red fluorescence emission.

### Microscopy and motor neuron observation

To quantify motor neuron abnormalities and other phenotypes below, we generated synchronized embryos for the different conditions tested. Corresponding adult male and female animals were separated the day before mating to be merged in fresh water the next day. Animals were monitored and left to mate for 1 h. The resulting embryos were considered ‘synchronized’ and collected in E3 medium without methylene blue. When appropriate, the embryos were injected with *hsa-SMN1* mRNA (injection in yolk of one-cell stage). The day of analysis, animals were dechorionated if required, anaesthetized in tricaine and observed sideways under a fluorescent microscope, unless stated otherwise. Only CaP-axon projection abnormalities were scored. One point was attributed to every motor axon that showed defects such as abnormal branching, abnormal length or absence of projection. Scores were compared in PRISM 9 using *t*-tests.

### Survival assay

Synchronised embryos were collected in E3 medium. When appropriate, the embryos were injected with human *SMN1* mRNA. Animals were sorted at 1 dpf based on their fluorescence profile and cultured in petri dishes for monitoring until 10 dpf. Paramecia were added to the medium starting from 6 dpf and animals were transferred into beakers starting from 10 dpf. Embryos/larvae were then counted each day to record death ratios over 20 days.

### *hsa-SMN1* mRNA production

*hsa-SMN1* mRNA was produced as previously described (5). Briefly, pCS2+:hsaSMN1 was linearized with NotI, purified and used with a mMESSAGE mMACHINE SP6 transcription kit (Ambion), following the manufacturer’s protocol. *hsa-SMN1* mRNA was purified using a MEGA clear kit (Ambion) following the manufacturer’s protocol, aliquoted and stored at −80°C. *hsa-SMN1* RNA (250 pg) was injected into the yolk of one-cell-stage embryos when required.

### Behavioural experiments and drug screening

Behavioural analysis was performed using the Zebrabox (Viewpoint) following the manufacturer’s instructions. Zebrafish larvae were distributed in 24-well plates filled with 500 μl of E3 medium (one larva per well, ± Riluzole as described, or DMSO 1%). Following the plates setup, all samples were placed at 28°C for a minimum of 1 h prior to run the analysis. The assays consisted of automatic recording of the larval swimming tracks/behaviour during 16 min (or stated otherwise) and under alternating phases of 4 min light and 4 min darkness. At the end of the experiment, each larva was monitored to exclude from the data potential dead animals. Data were processed using Excel/Prism.

### Cell-specific experiments

To test our material for cell-specific experiments, we generated an anti-*smn1* and anti-*insm1a* motoneuron-specific and anti-*dmd* muscle-specific construct. For anti-*dmd*, we combined p5E-503unc (p5E), 010_pME-mKate2 (pME) and 394_pDestTol2pA2 with 96-p3E-INTRON_*dmd1234* (Supplementary Figure S6) to generate the final injectable construct 503unc: mKate_DMD-1234. For anti-*smn1* and anti-*insm1a*, we combined 106_p5E-HB9 (p5E), 010_pME-mKate2 (pME) and 394_pDestTol2pA2 (Destination) with either 61_p3E_INTRON_*smn1-4141* or 97-p3E-INTRON_*insm1a123456* (Supplementary Figure S8) to generate the final injectable constructs HB9_mKate_smn4141 and HB9: mKate_insm1a-123456. We then injected different concentrations as described (± transposase) within the cell of one-cell stage wt or tg(MN:GFP) embryos. Control transgenes share the same cloning strategy and use a control p3E construct 72-p3E-INTRONcontrol1234, expressing 4× synthetic miRNAs without target/match in the genome as previously described (6). We analysed the animals from 1 to 6 dpf to assess toxicity, fluorescent expression, motor neuron development and muscle defects as presented in the manuscript. Data were exported and processed using Excel/Prism.

### Muscle fibres confocal observation

Prior to imaging, animals were anaesthetised with tricaine, embedded into 1.5% low-melting-agarose (LMT) and mounted (lateral view) in mater dishes. Animals were observed/recorded at 3 and 4 dpf to track mKate expression and analyse muscle fibre anatomy/integrity. Five minutes before recording (using an Olympus FV3000 confocal microscope), the media in the mater dishes were replaced to remove tricaine. Anaesthetic application and removal have been previously described to exacerbate muscle detachments in dystrophic mutants (17).

## Results

### Generation of an optimized kit for both RNAi and endogenous/natural miRNA expression

We first sought to develop a technology where strong and robust expression of our selectable fluorescent marker is able to occur together with multiple concatenated *pri-miRs*. We hypothesized that the RNAi expression cassette could be incorporated into an intronic sequence, leading to its splicing during mRNA maturation. This would result in a mature mRNA encoding the fluorescent marker terminated by a polyA tail alongside the spliced RNAi cassette(s) (Figure 1 and Supplementary Figure S1). Supporting this rationale, nearly half of the known human endogenous miRNAs are encoded within the introns of protein-coding genes (18). We reviewed the literature to find an appropriate sequence to use and selected the rabbit β-globin intron that has been demonstrated multiple times to be efficiently spliced in zebrafish while at the same time providing a significant increase in protein translation/expression (11,19,20). We assembled this sequence followed by an SV40 polyA signal within a Tol2kit empty p3E-vector carrying a kanamycin resistance cassette. Since the kanamycin resistance gene possesses a BsmBI restriction site that is incompatible with RNAi cassette assembly, we mutated this site so the final plasmid, named p3E-IntronRNAi (Addgene #163380, also called p3E-IntronMIR), would present only the two BsmBI sites required to introduce the synthetic *pre-miRs* (Figure 1A, and method section) (5). Note that the RNAi cassette is surrounded by unique restriction sites (BamHI-BglII/XhoI) enabling straightforward and endless chaining (Supplementary Figure S2).

Finally, to enable the expression and the study of endogenous miRNA, one can easily use the present BamHI/BglII sites to swap the present RNAi cassette with an amplified human or zebrafish *pri-miRNA*, surrounded by similar sites for keeping chaining versatility (Figure 1). One can either order these *pri-miRNA* as synthetic double-stranded DNA fragments or amplify them from the respective genomes using compatible oligos.

### Validation of the intron-based miR-delivery and RNAi approach

**Figure 3.**
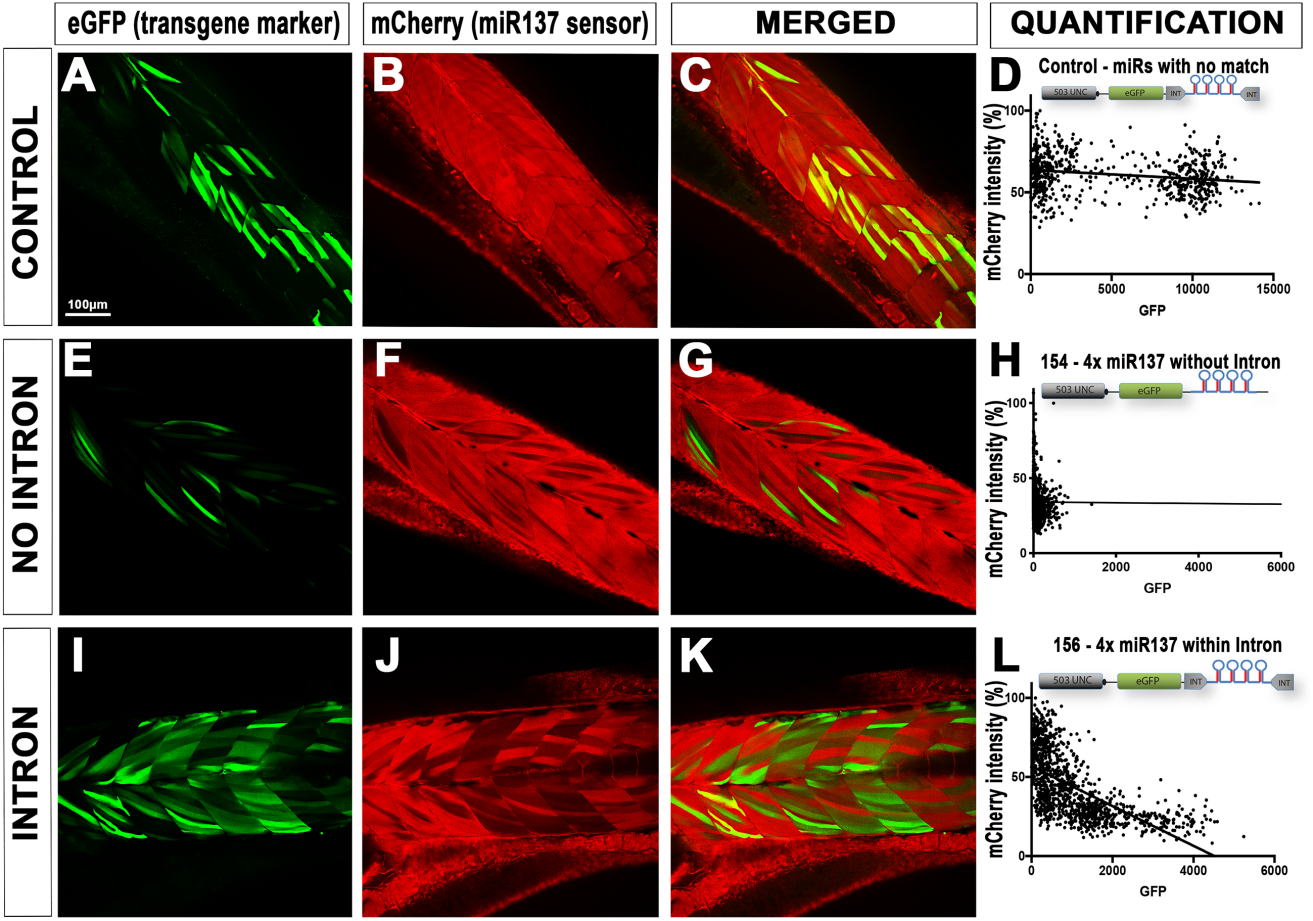
Validation of intron based RNAi approach on rescuing co-expression of fluorescent markers. (**A**–**D**), (**E**–**H**) and (**I**–**L**) Heterozygous Tg(ubi:mCherry:SPmiR137) embryos with ubiquitous mCherry expression were injected with Tol2 transposase and 50 pg of 4× anti-miR137 miRNAs construct without intron (plasmid 154) and with intron (plasmid 156), including a control (plasmid 157). A minimum of five larvae were imaged at 4 dpf using a confocal microscope to correlate the presence of 4× anti-miR137 expressing eGFP (with and without intron) to decrease/absence of mCherry fluorescence. Pixel intensities (grey values) of green versus red fluorescence (presented as a percentage) plotted in graphs H demonstrate no correlation of fluorescence in embryos injected with plasmid 154, similar to control 157. Comparatively, a linear correlation of green versus red fluorescence (**L**) is evidenced in embryos injected with plasmid with intron (plasmid 156).

To both test that (i) the miR/RNAi-delivery cassette is still functional when placed within an intron and (ii) that several *pri-miR* could now be chained without compromising the stability/co-expression of a fluorescent marker, we first conducted transient experiments. We took advantage of a transgenic line already available in the laboratory, i.e. tg(ubi:mCherry:SP137), which ubiquitously expresses an mRNA encoding mCherry and a custom 3′UTR acting as a sensor for endogenous and/or synthetic miR-137 (12). This line presents strong and homogeneous ubiquitous red fluorescent expression. We designed a synthetic *pri-miR* directed against this transgene 3′UTR. As presented in Figure 1 and in the method section, we first built a single-repeat 1×-RNAi cassette by inserting a custom *pre-miR137* into both the miR-delivery plasmids with or without intron. Following a traditional four component gateway LR reaction (Supplementary Figure S2B), we then generated two Tol2 1×-RNAi-transgenes encoding an eGFP and RNAi cassettes (with or without intron) under the control of a striated muscle-specific promoter (503unc) (6), respectively named 503unc:eGFP_RNAi_1×_miR137 (or 153) and 503unc:eGFP_RNAi_INTRON_1×_miR137 (or 155). A control transgene has also been generated using a previously developed RNAi cassette encoding non-targeting miRs (6). Those plasmids were injected along with transposase into tg(ubi:mCherry:SP137) as presented in Supplementary Figure S4. If functional, excepting for the control, the injected transgenes should lead to the co-expression of both eGFP and the designed synthetic-miR137 directed against the integrated ubi:mCherry:SP137 transgene, silencing its expression cell-specifically in striated muscle cells. At 4 dpf, we observed that all transgenes led to detectable GFP expression evidenced by mosaic green fluorescence, as expected for a transient DNA injection (Figure 2). All transgenes but the control also led to mosaic loss of mCherry expression, with a negative correlation/overlap with eGFP expression (Figure 2D, H and L), demonstrating proper cell-specific silencing of the targeted transgene and, thereby, validating that the presence of the intronic sequence did not interference with the maturation of the designed synthetic miRNAs (Figure 2). Second, we tested if the intron would help improving/rescuing the stability of the co-marker expression upon chaining several *pri-miR*/RNAi cassettes. Following a similar cloning strategy but chaining 4× *pri-miR137* together, we generated two new transgenes named 503unc:eGFP_RNAi_4×_miR137 (or 154) and 503unc:eGFP_RNAi_INTRON_4×_miR137 (or 156) (Supplementary Figure S2). As observed for the 1×-constructs, both plasmids successfully triggered eGFP expression and mosaic decrease/extinction of red fluorescence, demonstrating effective transcription of the transgenes and proper maturation of the associated *miR137* miRNAs (Figure 3). However, the 4×-*pri-miR* construct without intron triggered loss of red fluorescence that was not associated with eGFP expression. This observation was quantitatively confirmed as presented in Figure 3I, i.e. decrease of red fluorescence did not negatively correlate with green fluorescence emission, demonstrating that eGFP expression can be lost when several *pri-miRs* are chained. In contrast, when the 4× chained *pri-miRs* were embedded into the β-globin intron, one could still observe a negative correlation between loss/decrease of red fluorescence and presence/increase of green fluorescence, demonstrating that (i) the *pri-miR* repetitions no longer interfered with the expression of the marker cassette (in this case eGFP) and (ii) the presence/intensity of the fluorescent marker reflects the level of miR-expression. These conclusions were also supported by the transgenic/stable experiments presented below (Table 1).

**Table 1.** Generation of conditional *smn1*-LOF transgenic lines based on anti-*smn1* RNAi cassettes incorporating an intronic sequence or not

Responder lines generation	Number of selected F0 embryos	Number of F0 adult	Number of positive F0 founders	Selected F1 lines	Relative red brightness (NO iCre - unfloxed)	Relative blue brightness (+iCre - floxed)
tg(ubi:loxBFP: dsRED:miRsmn1-4141) Transgene without intron	50	38	14	#1	10	0
				#2	7	0
				#3	6	0
				#4	6	0
				#5	6	0
				#6	6	0
**tg(ubi:loxBFP:mKate: INTRON_miRsmn1-4141) Transgene with intron**	85	52	11	#1 or tg(floxSMN)	10	10
				#2	7	9 (Mosaic)
				#3	7	8
				#4	6	7
				#5	6	5
				#6	4	6

This table presents the number of animals screened/selected post-injection as well as the number of founders identified. All the founders have been compared and ranked based on the relative BFP expression/brightness of their progeny, with a score of 10 for the brightest. Only the six brightest lines were conserved and outcrossed with tg(ubi:iCre) to evaluate the conditional system efficiency and the associated red/mKate fluorescence. Note that none of the lines generated without an intronic sequence showed any detectable blue fluorescence in the presence of iCre.

### Generation of a robust inducible miRNA-expressing and RNAi system

Following the optimisation and validation of the new miR-delivery/RNAi cassette, we worked on establishing a robust inducible system. We previously used the Gal4/UAS technology but experienced important drawbacks that strongly hampered the flexibility and potential of this system (6). We experienced (i) random and strong methylation-induced silencing of the responder/miR-delivery transgenes, generating heterogeneous results difficult to analyse and (ii) toxicity problems with ubiquitous expression of Gal4. That being said, one advantage of the Gal4/UAS system was its ability to trigger strong expression of the responder/miR-delivery cassettes, thereby limiting the need to use long concatemers to achieve potent knockdown (6). Nonetheless, the problem of ubiquitous expression combined with random silencing generated too great limitations for both our basic research and our drug discovery programs. We thereby worked at establishing a more reliable conditional system for which we introduced the Cre/lox system (21).

**Figure 4.**
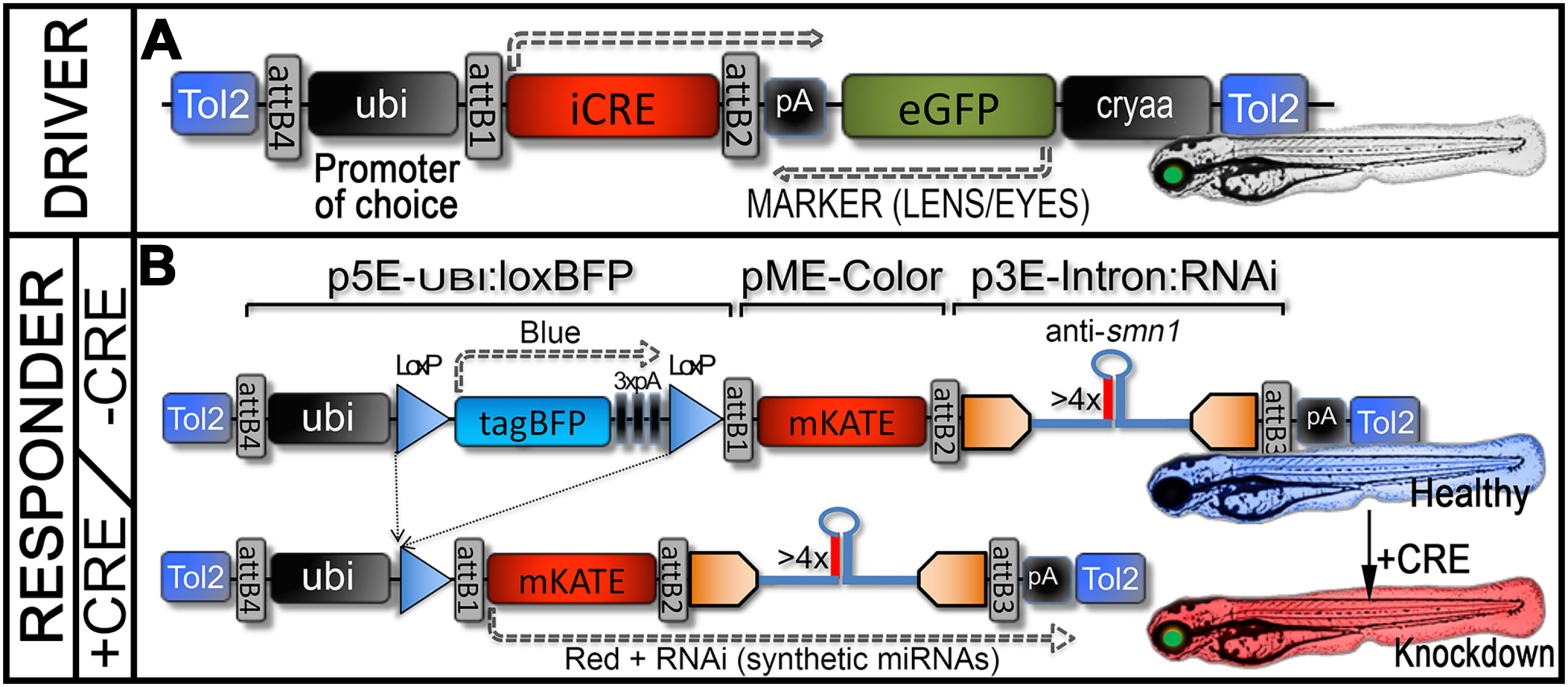
Conditional Cre/Lox RNAi genetic system. (**A**) Schematic representation of the driver transgene, with here a ubiquitous promoter, named ubi:iCre. Tissue-specific promoters can obviously be used. (**B**) Schematic representation of the silent/conditional gene-silencing responder transgene. In the absence of iCRE, only BFP will be expressed and the animal will remain unaffected by the RNAi cassette, thereby could be maintained without special care. In the presence of iCre, genetic recombination will lead to excision of the BFP cassette and expression of mKate along with the miRNAs of choice; with here 4× anti-*smn1* miRNAs, resulting in potent zebrafish *smn1* knockdown. This recombination is irreversible.

This alternative inducible genetic system is also based on driver and responder transgenes (Figure 4). The driver transgene/line is used to express Cre-recombinase in the desired tissue(s) and/or at the desired time-points in order to trigger Lox-recombination of the responder transgene(s); leading to either sequence deletion or inversion depending on the Lox-design/orientation. To establish the driver transgenes and lines used in this study, instead of using the Cre-recombinase commonly used in the zebrafish community, we incorporated the codon-improved Cre (iCre) into a Multisite Gateway/Tol2-kit compatible pME (i.e. pME-iCre, addgene #171792) (13), which is predicted to reduce silencing and increase efficiency overall (13,22).

Furthermore, we built destination clones with ubiquitous, muscle or pan-neuronal promoters [Ubiquitin, 503unc or HuC promoter respectively (6,16,23)], along with a marker cassette encoding eGFP under the control of a crystallin promoter (green expression in lens/eyes for identifying transgenic carriers) (Figure 4A). The corresponding driver transgenic lines have been generated through a traditional Tol2-transgenesis process, as described in the method section. Animals were selected and maintained thanks to eGFP expression in their eyes/lens.

To establish the conditional RNAi-responder transgenes, taking inspiration from the (ubi:Switch) system used for cell lineage (15), we built a genetic system allowing the integration of the miR-delivery/RNAi cassette in a silent state, and activable upon expression/presence of iCre (Figure 4B). For this, we built a p5E:ubi:lox:tagBFP:lox that can be recombined with any additional colour/marker (in this case mKate; red fluorescence) and any miR-delivery/RNAi cassette of choice. In practice, this system allows us to generate asymptomatic/silent-RNAi responder carriers expressing BFP ubiquitously that can be activated upon expression of Cre/iCre. In the presence of Cre/iCre, the BFP cassette would be floxed/excised triggering expression of mKate along with the miR-delivery/RNAi cassette, meaning the animals would turn from healthy/unaffected blue fluorescent to knockdown red fluorescent larvae. Additionally, this new system eases the selection of lines with strong expression of the RNAi cassette without having to deal with negative selection pressure (due to the potential phenotypic effect of the associated knockdown). Indeed, one can now easily screen animals based on BFP expression (blue fluorescence) to select carriers with strong transcriptional activity.

### Proof-of-principle and generation of a versatile model of spinal muscular atrophy

To validate the robustness of our inducible system, we went on to establish a conditional model of SMA. Having previously validated an anti-*smn1* RNAi cassette presenting 4× *pri-miR* repeats (named miRsmn1-4141) (5). We used this material to build two anti-*smn1* responder constructs/transgenes with similar organisation as presented in Figure 4B (one incorporating the intron and one without). Both constructs are similar but one presents the 4× RNAi block embedded into the aforementioned p3E-IntronRNAi. We named these anti-*smn1* transgenes ubi:loxBFP:dsRED:miRsmn1-4141 and ubi:loxBFP:mKate:INTRON_miRsmn1-4141. Both were injected at one cell-stage of tg(MN:GFP) embryos (transgenic line expressing eGFP in motor neurons) (23) along with transposase mRNA for random genomic integration. Fifty F0 embryos were screened based on visible tagBFP expression and raised until adult stage. Adults F0 were outcrossed against wt in order to identify founders that successfully transmitted the injected transgene. Fourteen (14) founders out of 38 F0 adult screened adults were identified for ubi:loxBFP:dsRED:miRsmn1-4141 (no intron) and 11 out of 52 for ubi:loxBFP:mKate:INTRON_miRsmn1-4141 (intron) (Table 1). We further compared and ranked those founders on a scale of 1–10 based on the BFP brightness/expression observed in their progeny (the brighter the higher the score). The best six founders for each transgene were isolated and outcrossed with tg(MN:GFP), with resulting clutches screened for embryos displaying blue fluorescence that were raised to adulthood to generate stable F1 responder lines.

Those F1 animals were further outcrossed with tg(ubi:iCre) to evaluate the efficiency of this new conditional system. While all tg(ubi:loxBFP:mKate:INTRON_miRsmn1-4141 -intron-) displayed bright red fluorescence (Table 1), demonstrating effective Lox-recombination of the tag-BFP cassette (Figure 4B), none of the tg(ubi:loxBFP:dsRED:miRsmn1-4141 -no intron-) showed any detectable red fluorescence, suggesting proper recombination of the tagBFP cassette but no observable activation of the marker/RNAi block. Remarkably, although floxed tg(ubi:loxBFP:dsRED:miRsmn1-4141 -no intron-) did not display any red fluorescence, they did develop obvious traits of SMA, such as loss of motor function and premature death. Considering these preliminary observations, we hypothesized that the anti-*smn1* miRNAs were properly expressed but their processing/maturation hampered the expression of the red fluorescent marker, and decided not to progress these lines. On the contrary, all floxed tg(ubi:loxBFP:mKate:INTRON_miRsmn1-4141) displayed red fluorescence with brightness ranking similar to their unfloxed blue fluorescent counterparts, i.e. the brightest unfloxed blue fluorescent lines/embryos translated to the brightest floxed red fluorescent samples (Table 1). We then selected the brightest F1 line, tg(ubi:loxBFP:mKate:INTRON_miRsmn1-4141)#1, hereafter named tg(loxSMN), to further analyze its ability to generate SMA-like larvae.

**Figure 5.**
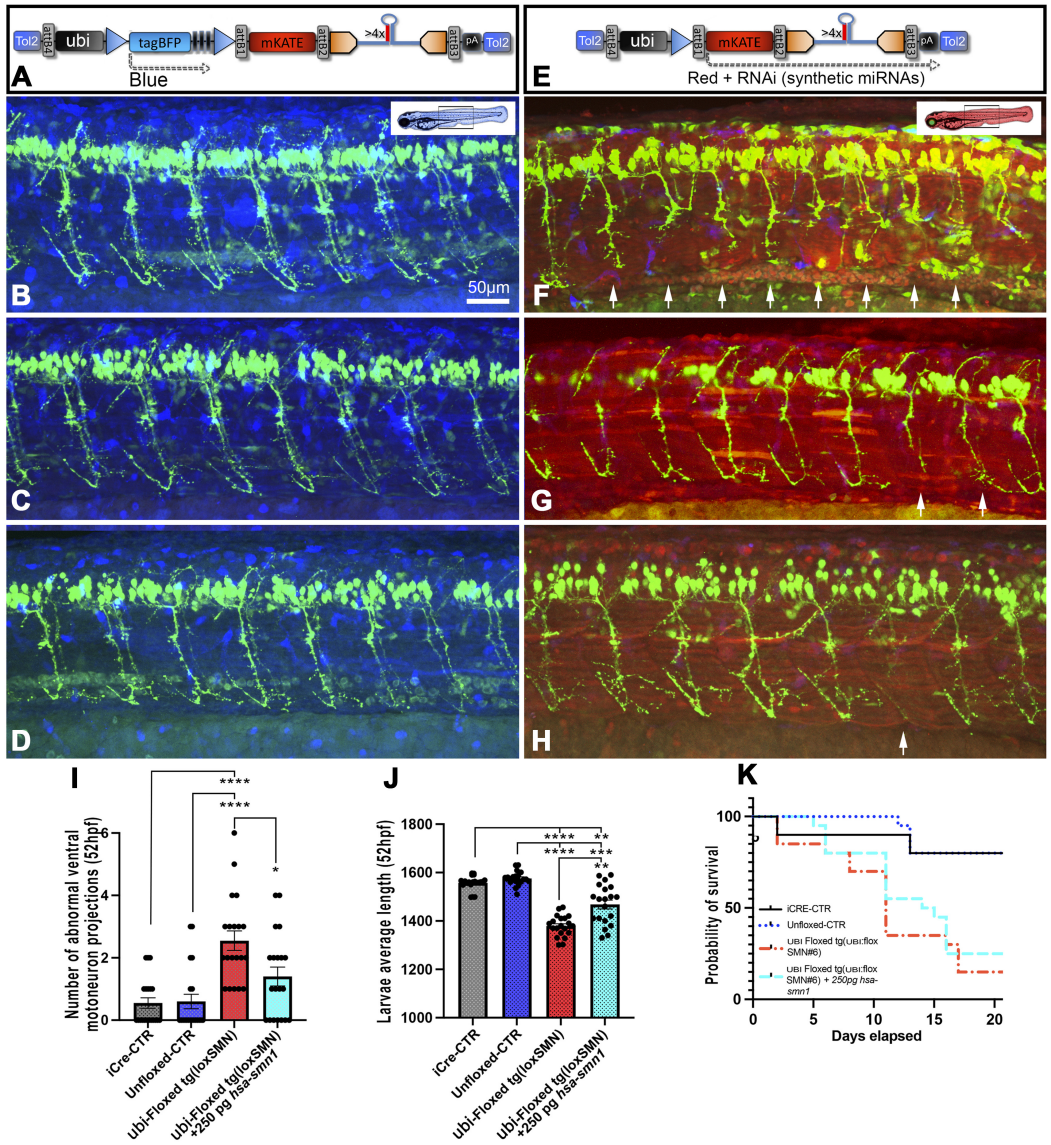
Representative snapshots and phenotypic analysis of 50 hpf zebrafish larvae with unfloxed (A–D) or floxed (E–H) integrated RNAi transgene. (**A**–**D**) In the absence of iCRE, the transgene remains unfloxed triggering BFP expression and not inducing any significant developmental defect in the analyzed embryos. (**E**–**H**) In the presence of iCRE, BFP is excised and mKate expressed along with anti-*smn1* miRNAs, triggering SMA-hallmarks such as motoneuron defects. Images represent standard deviation projections from confocal z-stack acquisitions using three channels (merged) to detect eGFP (green, motoneurons), tagBFP (blue, ubiquitous expression) and mKate (red, ubiquitous expression). White arrows indicate abnormal CaP motoneurons. (**I**) Number of ventral motoneuron (CaP) abnormalities observed per side of 50 hpf larvae. ubi-floxed tg(loxSMN) animals were also injected with *hsa-SMN1* mRNA to test the specificity of the observed phenotype. (**J**) Animal size comparison at 52 hpf. (**K**) Survival assay (Kaplan Meier) for the different lines and conditions tested. Means of 20 larvae ± SEM.

We previously showed that *smn1*-LOF triggers motor neuron developmental defects, progressive loss of motor function and premature death as observed in patients (5). We therefore tested if we were able to reproduce those phenotypes using this new system and generate a versatile model for research. To analyze those phenotypes, we performed a series of experiments in which we outcrossed tg(loxSMN) with tg(ubi:iCre) and sorted embryos based on their fluorescence profile. Two controls were used, one with unfloxed embryos (BFP positive, mKate negative), named ‘Unfloxed-CTR’, and another from a cross between tg(MN:GFP) and tg(ubi:iCre), named ‘iCre-CTR’. In terms of motoneuron development, we found that floxed tg(loxSMN) developed significant abnormal ventral CaP projections with 2.55(±1.39) at 50 hours-post-fertilisation (hpf) when compared to controls iCre-CTR (0.55 ± 0.75) and Unfloxed-CTR (0.6 ± 0.75) (Figure 5A–I). Demonstrating the specificity of this phenotype, injection of 250 pg of human *SMN1* mRNA significantly reduced these defects with only 1.4(±1.35) abnormal CaP projection per side of animal. Floxed tg(loxSMN) also presented with a slight growth retardation with an average length (1379 ± 45), significantly shorter than the controls iCre-CTR (1561 ± 26) and Unfloxed-CTR (1580 ± 21) (Figure 5I). Injection of human *SMN1* mRNA also significantly rescued this growth retardation with an average of 1467(±20). These defects also translated into a progressive loss of motor function as presented in Supplementary Figure S5, as well as in premature death (Figure 5K). Indeed, while 80% or more of both controls survived past the survey of 20 dpf, only 15% of the sorted floxed tg(loxSMN) animals survived >20 dpf; and none surviving until adulthood. Injection of 250 pg of human *SMN1* mRNA did not rescue the premature death of the floxed tg(loxSMN), which was to be expected, as injected mRNA usually gets degraded by, or before, 5 days-post-injection/fertilization.

Together, these results demonstrate that our Cre/Lox miR-delivery/RNAi technology is effective and we successfully generated a conditional genetic system able to produce healthy RNAi-carriers that can be ‘ranked’ and easily maintained thanks to the fluorescence associated with the unfloxed state. We showed that, while asymptomatic/silent, these lines can be easily activated to recapitulate strong human disease hallmarks, such as the SMA defects and premature death presented here. It is noteworthy that these silent RNAi-responder transgenics can be activated not only by transgenic cross with Cre-driver lines, but also by injection of either Cre/iCre mRNA or Cre protein (13). In a side study, we have already worked at defining the recombinant effect of different Cre mRNAs and protein versions as well as dosage effects for controlling this new genetic miR-delivery system (13).

### Versatile genetic system for drug discovery or large-scale experiments

Drug discovery using the zebrafish animal model is becoming popular. However, it is often difficult to develop models of human diseases that would be compatible with large-scale (high throughput/content) screening. First, one needs to establish a model that develops a robust and early phenotype, i.e. ideally taking place in the first 2–7 dpf. The presented floxed tg(loxSMN) develops progressive loss of motor function (Supplementary Figure S5A and S5B) and premature death (Figure 5K) that could be used as readouts in a screen. Animals start to demonstrate a decrease in their spontaneous swimming behaviour starting from 5 dpf and with a clear abnormal pattern at 6 dpf, as demonstrated in Supplementary Figure S5A and S5B. Our assay consisted of an automatic record of the larvae swimming tracks for 16 min with alternating light and dark phases. In these conditions, while control iCre-CTR swam for an average of 271mm (±107mm), floxed tg(loxSMN) swam significantly less with an average of 121mm (±91mm). Although relatively heterogeneous, this readout could be used to establish a screen, looking for compounds improving/rescuing those motor defects. Second, and most importantly, to be able to run a drug screen or a large-scale experiment, one should be able to produce a large number of affected animals that can be identified before the assays or readouts. For example, when one uses mutants with early phenotype, usually only heterozygotes could be maintained for breeding with only 25% of the resulting embryos producing early phenotype (Supplementary Figure S6A). One has to either be able to genotype the embryos before the experiments, or deal with 75% data points that would bias the readouts/results. In contrast, here, one can sort the embryos based on their fluorescent expression prior to the screen (Supplementary Figure S6B and S6C). Moreover, we found that crossing female tg(ubi:iCre) drivers with male RNAi responders, led to 100% conversion of the RNAi lox cassette, whether the female tg(ubi:iCre) driver was heterozygous or homozygous, strongly facilitating the set-up of screening plates. We hypothesized that this was due to maternal deposition of iCre-mRNA and iCRE-protein into the embryos (13). Demonstrating the value of this genetic system and approach for drug screening, we tested serial dilutions of the neuroprotective drug Riluzole in 24-well plates, from 10 μM down to 10 nM. We found that 10 μM was lethal while 1 μM slightly improved motor functions of floxed tg(loxSMN) animals at 6 dpf (Supplementary Figure S5C). No further effect was observed on either motoneuron development or premature death, suggesting unfortunately a poor therapeutic outcome on the presented zebrafish SMA model.

### miR-delivery genetic system enabling tissue-specific studies

**Figure 6.**
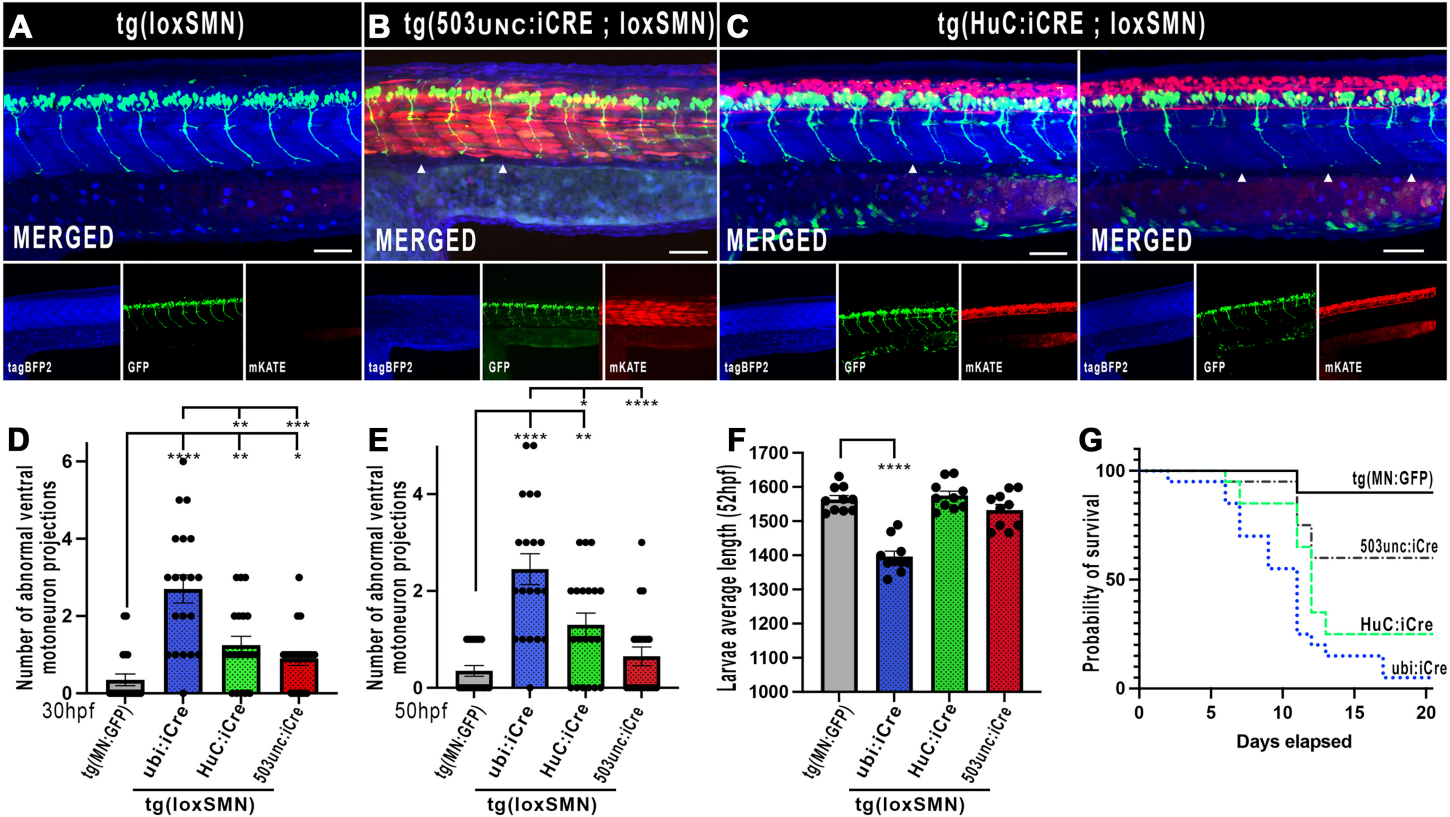
The RNAi Cre/Lox system allows tissue-specific gene(s) silencing. (**A**–**C**) Representative snapshots of unfloxed and tissue-specifically floxed animals. (**D**and **E**) Numbers of abnormal ventral motoneuron (CaP) observed per side of 30 hpf (A) and 50 hpf larvae (B). (**F**) Animal size comparison at 52 hpf (*n* = 10). (**G**) Survival assay (Kaplan Meier) for the different lines tested. ubi. Ubiquitous promoter. HuC, Pan Neuronal promoter. 503unc, Striated muscle promoter. Means of 20 larvae ± SEM.

We further tested the ability of the presented system to conduct tissue-specific studies. We generated transgenic lines using Tol2 iCre-transgenes designed to drive pan-neuronal- (HuC promoter) or striated muscle- (503unc promoter) expression. Each transgene co-expresses an eGFP marker under the control of a crystallin promoter, enabling straightforward tracking of the carriers and downstream lines. We generated and validated stable transgenic driver-lines and demonstrated that pan-neuronal Lox-recombination in tg(loxSMN) triggered motoneuron defects similar to the one observed in ubiquitous lines (Figure 6). Surprisingly, these defects were not as pronounced as in the ubiquitous condition, with only 1.3(±1.4) abnormal CaP branch at 50 hpf, significantly different from 2.45(±1.4) at 50 hpf in the ubiquitously floxed lines (Figure 6D and E). This difference may be explained, at least in part, by the fact that the gene *smn1* is ubiquitously expressed and that a certain amount of Smn protein should already be present in the early differentiated neurons before activation of the miR-mediated *smn1*-knockdown following iCre transcriptional activity. This feature could certainly represent a limitation of this technology when one desires to work tissue-specifically. In contrast, although striated muscle expression of the anti-*smn1* miRNAs triggered significant early motoneuron defects (0.9 ± 0.8 at 30 hpf), there was no further evidence of significant permanent motor neuron phenotype at later stage (0.65 ± 0.9 at 50 hpf). All conditions did significantly impact the survival of the animals (Figure 6G). However, it is notable that most 503unc-animal deaths happened at around 11 dpf and seem to be related to an absence of an inflated swim bladder. All in all, those data demonstrate the potential of this system to drive inducible and/or tissue-specific gene-silencing experiments. This controllable genetic system should also be very useful for microRNA basic research studies by allowing versatile control and expression of endogenous miRNA(s) of choices.

### Rapid cell-specific miR-delivery/RNAi studies

**Figure 7.**
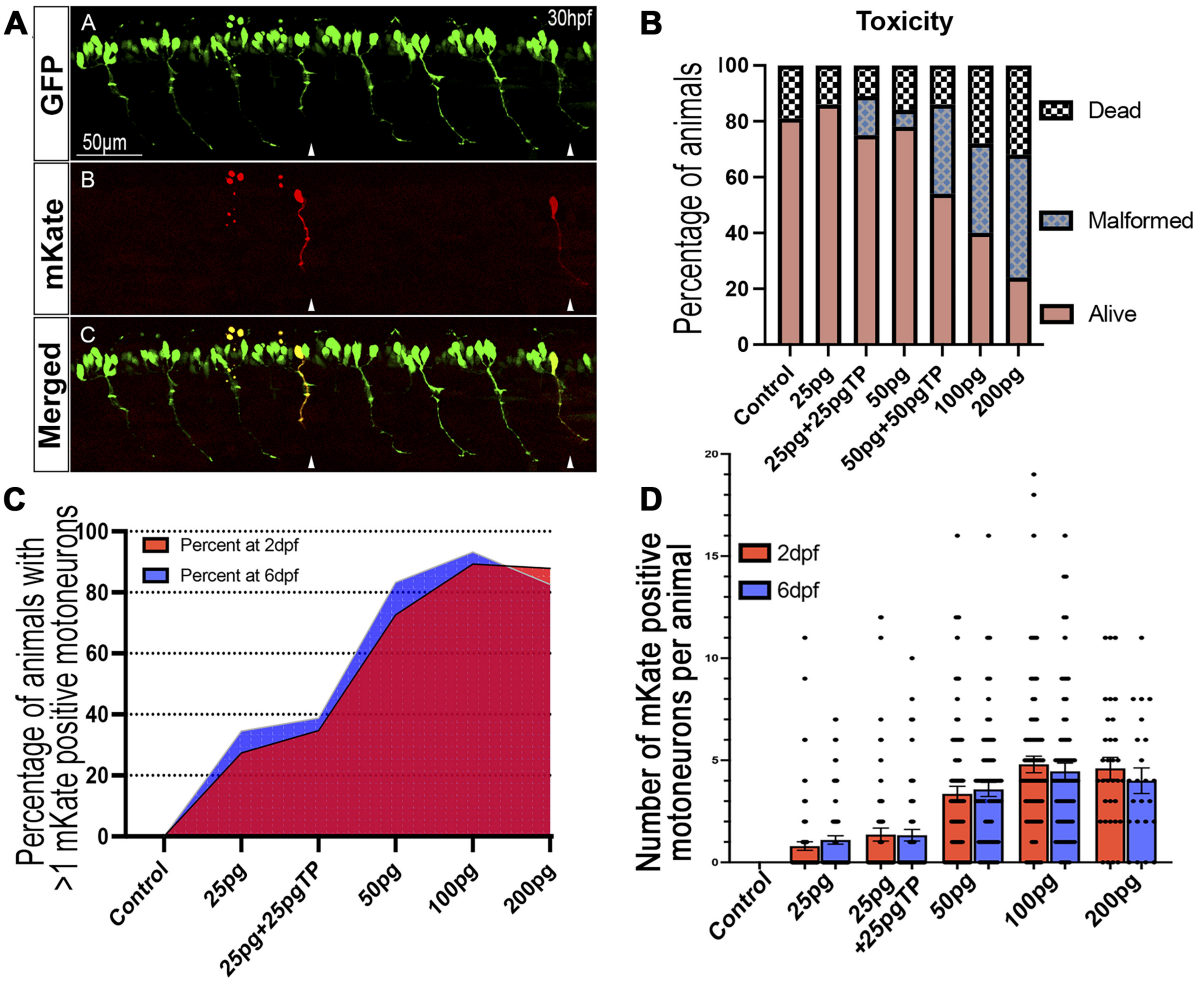
Cell-specific miR-delivery/RNAi in zebrafish does not require transposase. We tested the ability of the presented miR-delivery system to enable rapid cell-specific experiments. The approach is similar to the method conducted in Figures 2 and 3 but aimed at evaluating the effect of different injection mix. (**A**) Schematic example of the expression obtained following the injection of a motoneuron-specific miR-delivery construct (83_HB9_mKate_smn4141) within the cell of one-cell stage tg(MN:GFP) animals. Arrowhead indicating mKate positive motoneurons with positive transgene expression. (**B**) Titration/Toxicity test of Tol2-construct injections (83_HB9_mKate_smn4141) for triggering cell-specific expression. (**C**) Number (percentage) of animals presenting at least 1× mKate positive motoneuron. (**D**) Average number of mKate-positive CaP motoneurons per positive animal. Eighty embryos per condition have been analyzed (means ± SEM). These results suggest that a dose of 50 pg without transposase may be ideal for setting up cell-specific approaches and studies.

As presented in Figures 2 and 3 above, the ability to track the injected miR-delivery/RNAi transgenes opens the door to rapid mosaic cell-specific experiments. Following injection, each cell presenting transcriptional activity of the injected transgene will present fluorescence and could be compared to their non-expressing counterparts within the same animal. To optimize the injection mix for such an approach, we constructed a motoneuron-specific anti-*smn1* construct combining 106_p5E-HB9, 010_pME-mKate2 and 61_p3E_INTRON_*smn1-4141* with a 394_pDestTol2pA2 destination clone. The final construct was named 83_HB9_mKate_smn4141. We injected this construct in one-cell stage tg(MN:GFP) animals at different concentrations, ranging from 25 to 200 pg final, complexed or not with transposase (Figure 7). Our experiments suggest that a concentration of up to 50 pg of plasmid per cell is well tolerated while toxicity is observed starting from 100 pg onward (Figure 7B). We then quantified the number of animals presenting at least 1× mKate-positive CaP motoneuron (evidencing the activity of the miR-delivery transgene, Figure 7A arrowheads) and plotted the different percentage obtained in Figure 7C. Surprisingly, our data suggest that the addition of transposase (promoting transgene integration) did not seem to greatly impact the percentage of positive animals per injected batch (Figure 7C) or the average number of positive expressing cells per injected animal (Figure 7D). While 25 pg of plasmid alone led to a batch with 27.4% animals presenting at least 1 mKate-positive CaP -with 2.91 ± 2.73 positive CaP-, the addition of transposase mRNA (25 pg) did not dramatically change this ratio with only 34% of positive animals -with 3.92 ± 3.3 positive CaP-. However, the injection of 50 pg of plasmid alone led to 72.6% of positive animals presenting an average of 4.3 ± 3.2 positive CaP per positive animal. From 50 pg, the addition of transposase or a higher plasmid dose led to significant malformations that would interfere with the experiments (Figure 7B). Remarkably, the expression of the transgene (with or without transposase) was detectable long after injections with constant pattern at least up to 6 dpf (Figure 7D). Those results suggest that a dose of 50 pg without transposase would be a range of choice for conducting mosaic cell-specific experiments.

To test this approach further, we constructed two additional transgenes directed against the genes *dmd* or *insm1a*, and analyzed if these transgenes can reproduce part of the previously demonstrated mutant phenotypes, with early stochastic muscle defects/detachments for *dmd* (17,24,25) and abnormal CaP motor neuron development for *insm1a* (26). We deployed 4× repeats against *dmd* (Supplementary Figures S7 and S8) and 6× repeats against *insm1a* (Supplementary Figures S9 and S10). Confirming again the efficiency of the intronic sequence and constructs, mosaic expression of mKate could be robustly detected in the striated muscle fibres or motor neurons of the respectively injected embryos. For *dmd*, while no defect could be detected in the control animals, the anti-*dmd* transgene successfully recapitulated stochastic muscle defect/detachments observed in dystrophic mutant sapje(*dmd*) (17,24,25), clearly demonstrated by both birefringence (Supplementary Figure S8C) and confocal microscopy (Supplementary Figure S8D and S8E). During the confocal recording, we successfully captured muscle detachment in real time as presented in Supplementary Videos S01 and S02, events specifically reported in dystrophic animals (17). It is noteworthy that the animals developing detectable muscle defects also presented reduced swimming patterns at 5 dpf as evidenced by 24 min swimming recordings conducted with a Zebrabox Revolution (Supplementary Figure S8F). Similarly, motor neuron mosaic expression of anti-*insm1a* miRNAs successfully phenocopied some hallmarks of the respective *insm1a* mutant (26), with obvious impact on the axonal length and number of mKate-positive CaP motor neurons (Supplementary Figure S10C, S10D and S10E). However, in our experiments, those defects do not translate into motor function phenotype/reduction (Supplementary Figure S10F). This is not surprising as we demonstrated in Supplementary Figure S10D that injection of 50 pg leads to 4.3 ± 3.2 mKate-positive CaP motor neurons which may not be sufficient to affect the overall swimming ability of the animals, or not enough to be statistically detectable.

## Discussion/conclusion

We have previously demonstrated that miR-mediated gene knockdown could be a powerful tool for zebrafish genetics and that this technology has the potential to be a versatile complement to CRISPR/Cas9 approaches (27). Those approaches could also ease the studies of endogenous microRNA biology by offering a versatile genetic system for transgenic delivery. Unfortunately, those tools were suffering from important limitations that were hampering their potential. First, let alone multi-genic silencing, to achieve proper single-gene knockdown, multiple synthetic miRs must be expressed simultaneously, which compromises the co-expression of a fluorescent marker/tracker, strongly reducing the interest in this approach. Second, when knockdown of the gene(s) of interest triggers significant phenotype/defects, it is often hard to generate and maintain stable transgenic carriers with strong expression.

Here, we optimized this gene-silencing and miR-delivery approach and established a new and mature genetic system for zebrafish. Based on our analyses, this genetic system no longer suffers from limitations due to the processing of the synthetic miRs, and one can now chain multiple miR-delivery/RNAi cassettes without taking the risk of losing the coupled fluorescent tracker/marker. In theory, any combination of microRNAs can now be simultaneously expressed and quantitatively monitored via the associated fluorescence. Any gene of interest can now be targeted, and multi-gene knockdowns should also be easily achievable. Although this approach may not be able to surpass the knockout efficiency and reliability of the recent CRISPR/Cas9 methodologies, the ability to track cells, tissues and organisms during the analysis is of unique value and flexibility for research. In addition, we are delivering here a conditional genetic system that proves to be powerful, enabling the generation of healthy carriers that can now be easily maintained. Importantly, even when not activated, this system offers the advantage of enabling the selection and monitoring of transgenic lines/carriers with high transcriptional activity of the ‘silent’ miR-delivery/RNAi transgene, thanks to the BFP/blue expression (different sets of fluorescence markers could be used). Another important advantage is the ability to drive the activity of the miR-delivery/RNAi cassette spatiotemporally, opening the door to cell- and tissue-specific investigations, an approach that is still unmatched today with the zebrafish model. Moreover, the potentiality to either use transgenic Cre-driver or injectables (Cre mRNA or protein) (13) offers great versatility for the investigators, allowing, for instance, to mix different responder-RNAi without performing complicated and lengthy crosses. We also demonstrated that, using this method, one could easily produce a large number of embryos that would not require a pre-screen before the experiments (Figure 4 and Supplementary Figure S6), offering greater flexibility for research and large-scale experiments. All in all, this technology and methodologies should work synergistically with CRISPR/Cas9 approaches to ease functional genetics studies and complement it to better address questions associated with haploinsufficient conditions and/or when protein dosage is more important than total LOF. Last but not least, this method and material should be of high value to express and study microRNAs biology.

## Supplementary Material

gkaf004_Supplemental_Files

## Data Availability

The data underlying this article will be shared on reasonable request to the corresponding author.
