# Supplementary material for: Cre-Lox miRNA-delivery technology optimized for inducible microRNA and gene-silencing studies in zebrafish
Source: Nucleic Acids Res. 2025 Jan 20;53(2):gkaf004. doi: 10.1093/nar/gkaf004 (PMC11744099; doi:10.1093/nar/gkaf004)
Supplement: gkaf004_Supplemental_Files [file gkaf004_supplemental_files.zip › 2024_zebrafish_Cre_SMA_SUPP.pdf]

Supplemental material

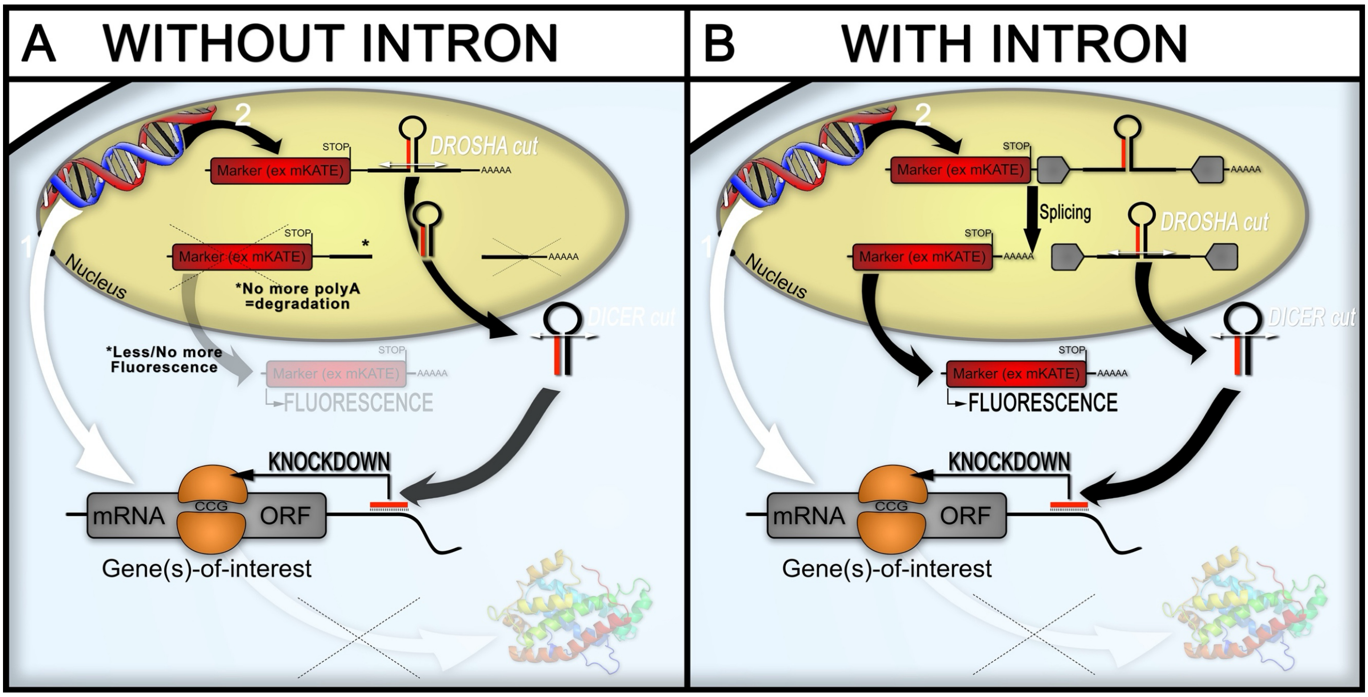

Figure S1. Schematic representation of transgenic miRNA-delivery technique in zebrafish and the benefit of using intronic sequence for proper processing. A, Without Intron, the miRNA-delivery cassette will be included in a mRNA along with a fluorescent marker. Unfortunately, the maturation of the miRNA will involve cuts that will lead to the denaturation of the mRNA due to the loss of its polyA tail. Consequently, the translation of the fluorescent marker and the maturation of the synthetic miRNA will work competitively, with the brightness of the marker not being proportional/correlated to the expression of the *miRs*. The fluorescence would be completely lost when using 3x or more *pri-miR* repeats. **B,** The introduction of an intronic sequence solves this limitation by inducing splicing of the *pri-miR* cassettes prior to Drosha cuts, leaving the host mRNA intact/stable and enabling co-expression of the attached fluorescent marker.

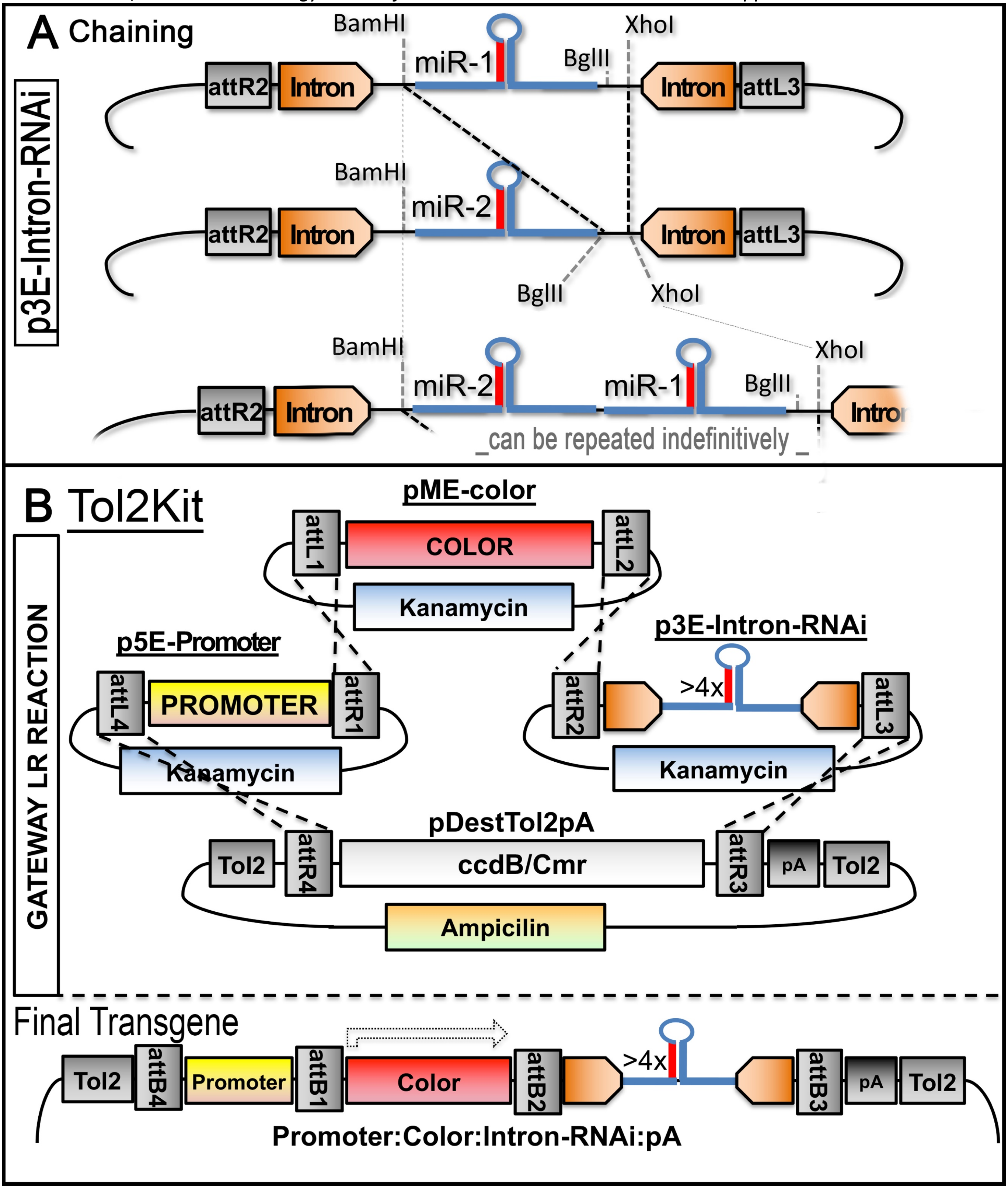

Figure S2. Schematic representation of p3E-Intron_miR_RNAi cloning procedures. A, The P3E plasmid is optimised for easily chained multiple *pri-miR* cassettes as concatemer using digestion reactions that can be repeated through unlimited rounds. **B,** Typical Tol2kit/multisite-Gateway reaction.

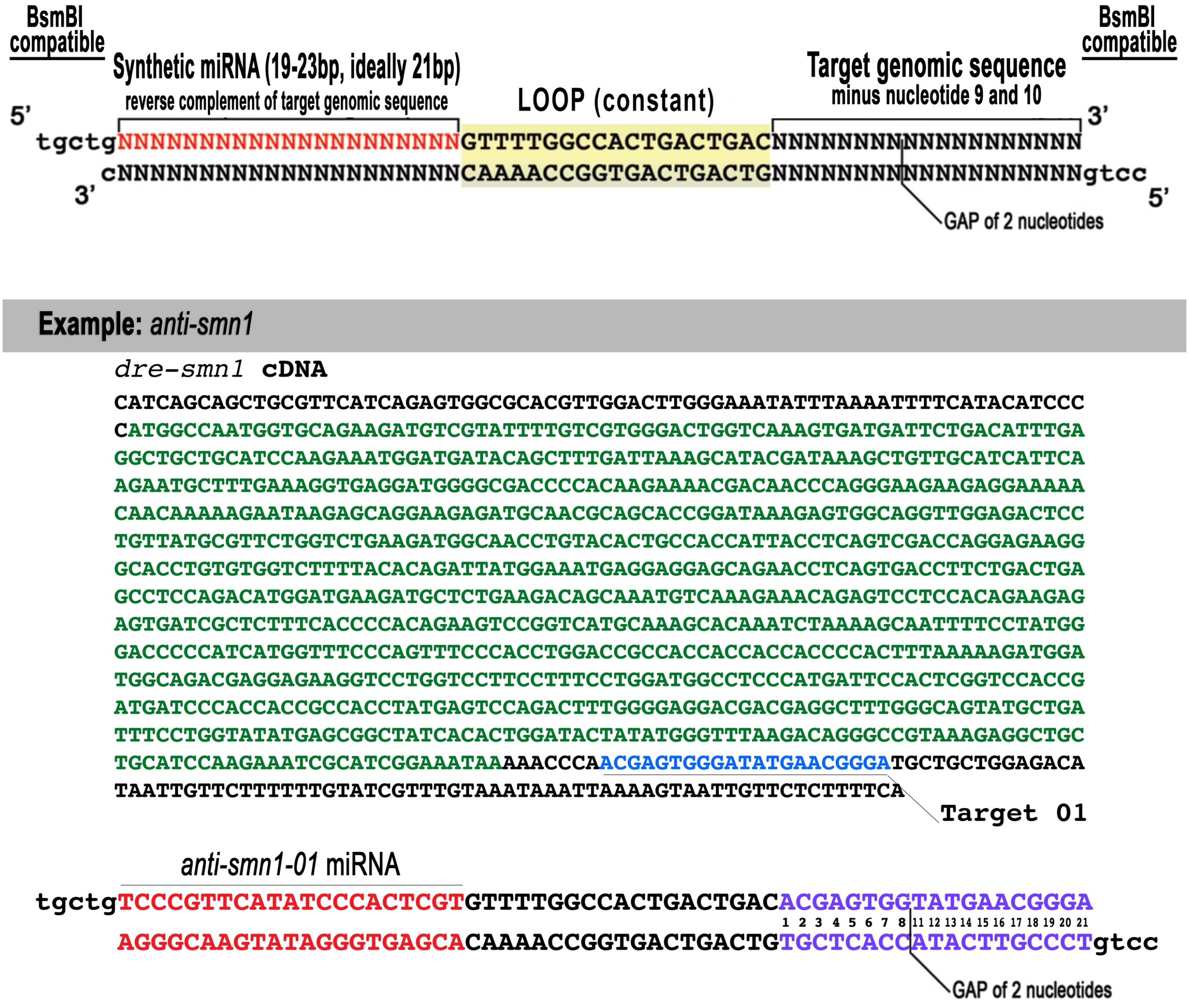

Figure S3. Synthetic *pri-miRNA* design. Top and bottom oligo to order for generating (by annealing) *pri-miR* to be inserted into BsmBI-digested p3E-IntronMIR / p3E-IntronRNAi. Sequence in red should be the perfect reverse complement of the DNA target sequence. See the example to target the gene *smn1* (CDS represented in green sequence and 5' and 3'UTR in black).

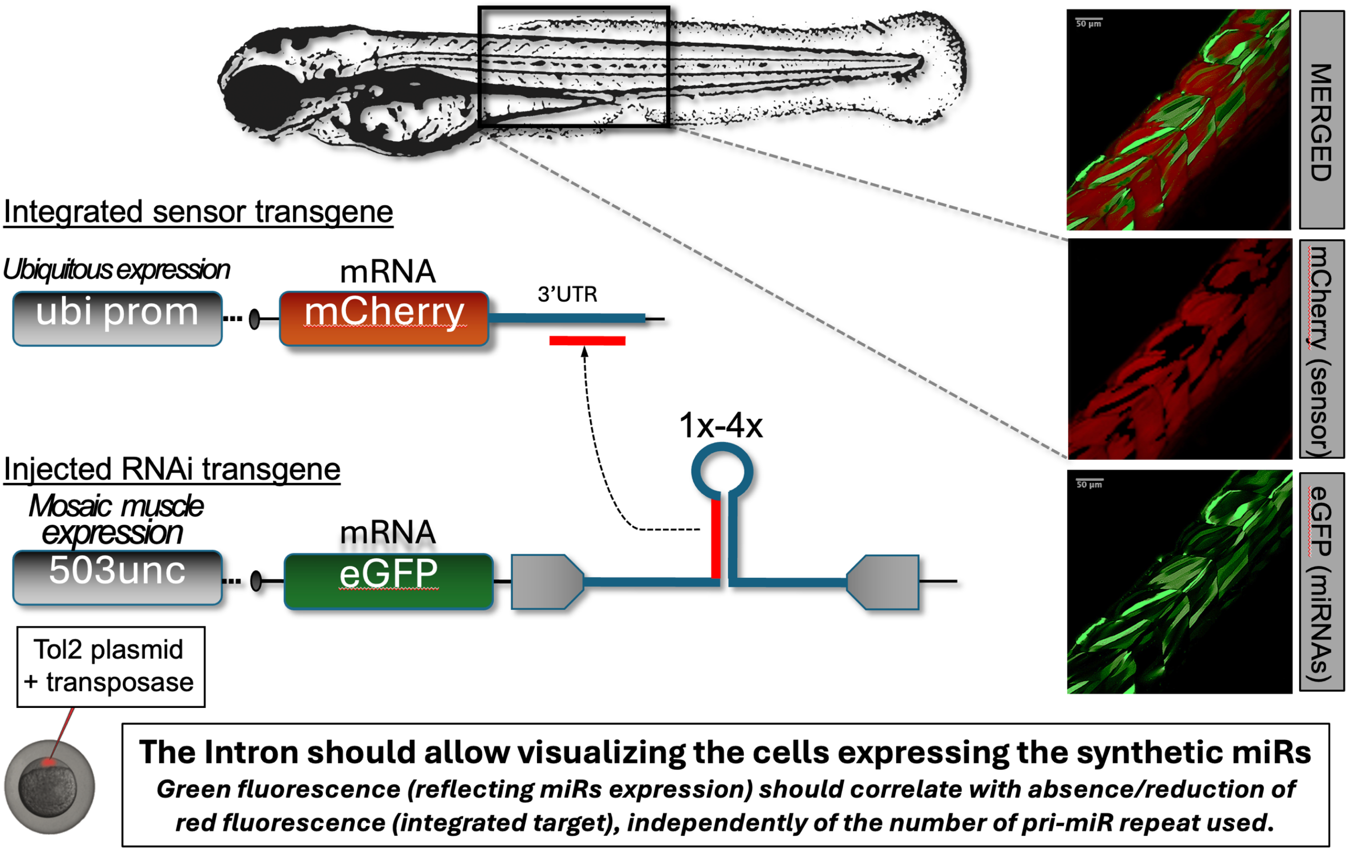

Figure S4: Schematic representation of the approach used to assess/validate the effect of the intronic sequence and outcome illustration example.

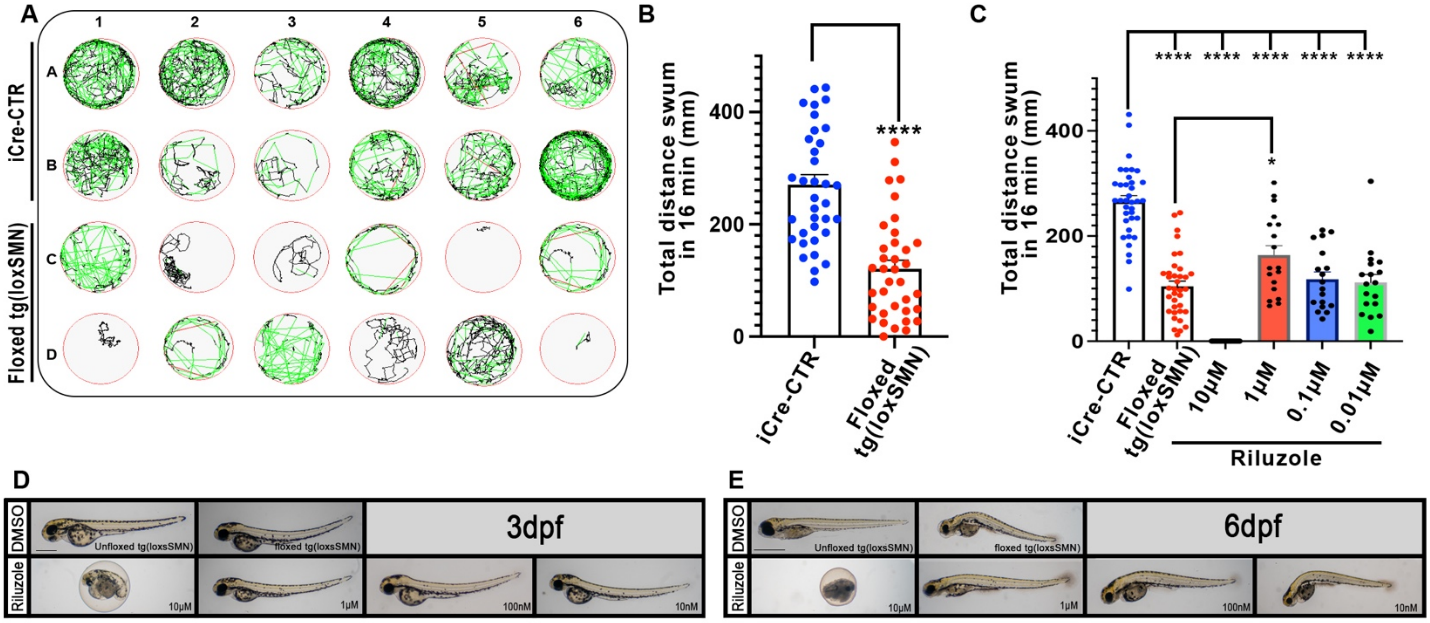

Figure S5. Conditional RNAi system suitable for large-scale experiments. Drug screening requires large number of "affected" samples that can be identified/genotyped prior to the screens. This could be a problem when one works with mutant lines. The presented iCre/Lox RNAi strategy offers the advantage of facilitating the generation of a large number of LOF-animals that can be identified prior to the screen thanks to their fluorescent profile (fig. S04). A, Swimming tracks of iCre-CTR and Floxed tg(loxSMN) after a 16min recording alternating 4min of light and dark phases. Black tracks represent slow speed (<2mm/sec), green tracks medium speed of (2-6mm/sec) and red tracks high speed (>6mm/sec). B, Average distance swum by 36 larvae throughout 16min alternating 4 minutes of light and 4 minutes of dark. C, Effect of Riluzole on the swimming behaviour of floxed tg(loxSMN) animal. Results are represented as means ±SEM with dots representing a single data point or well. D-E, Representative images of treated animals at 3dpf and 6dpf.

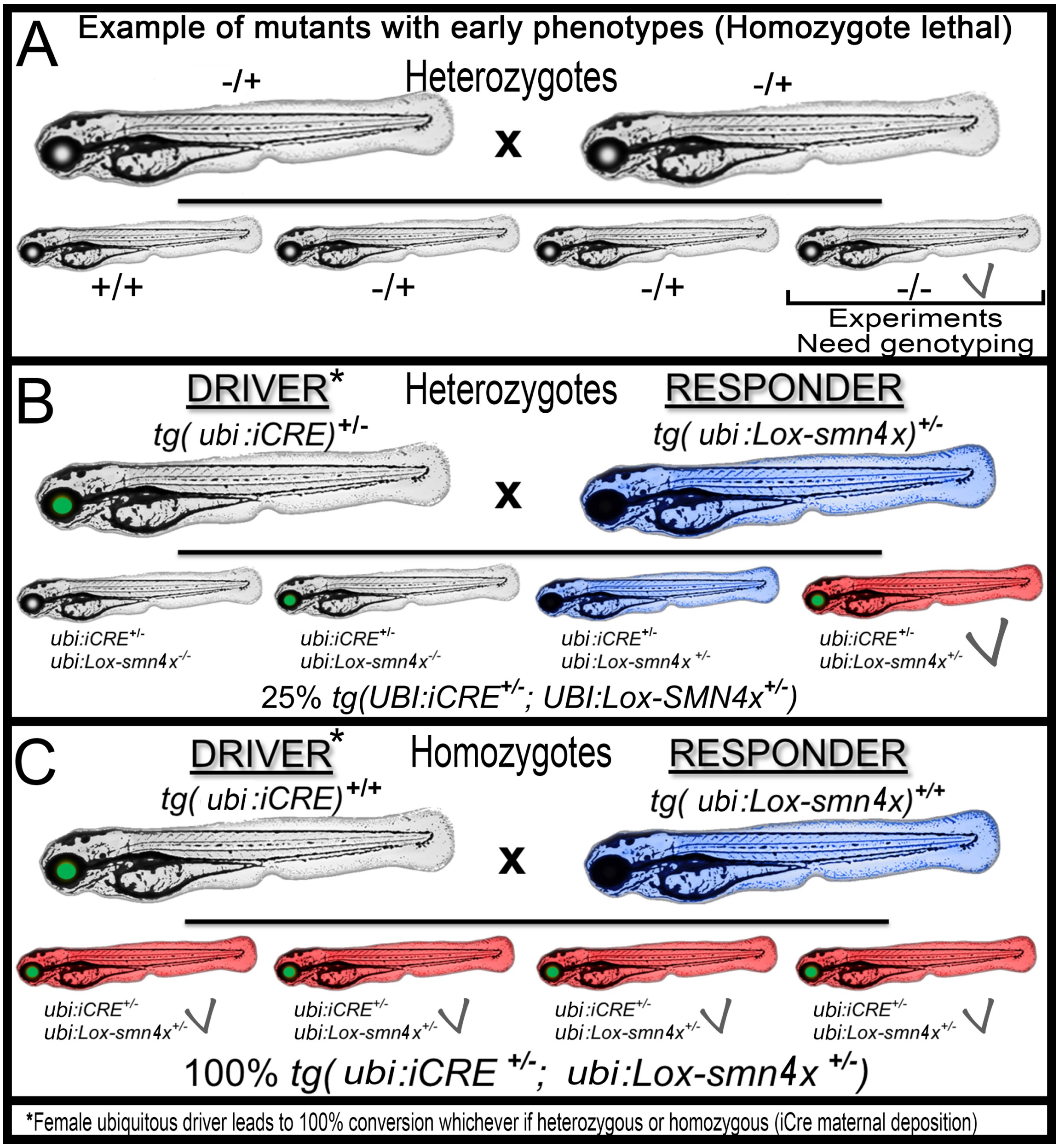

Figure S6: Illustration presenting crossing required to generate embryos for drug screening or large-scale experiments. A, Zebrafish disease models of human diseases are usually suitable for drug screening if they present early and strong phenotypes that can be analysed in multi-well plates. Unfortunately, for models based on mutations, these defects mean that the line can only be maintained in heterozygous state. To generate embryos for the screens, one should incross heterozygous, leading to only 25% of affected homozygous embryos that cannot be identified from their unaffected sibling before the end of the experiments; strongly hampering large-scale experiments. **B-C**, The presented Cre/Lox system can greatly facilitate the generation of affected animals as presented in the schematics. First, the animal could be easily identified before the screening procedure thanks their fluorescent profile. Secondly, and most importantly, this conditional system enables to generate 100% affected animals, strongly easing the experimental procedure. Working with transgenic responder lines leading to strong knockdown while presenting only one insertion is however important for the homogeneity of the screens. Note that female Cre-driver with maternal Cre-expression would lead to 100% conversion even in the absence of driver-transgene transmission.

A.

ENSDART00000170290.2

TCATTCACAAAAAATTCACTGCTGGATCTGAGGTTCAGTTTGTTCACAGGGCGTTGGTGAGGGC
TCCTTCCTTTTTCTGTTTATAAAATTGTAGGATTAAAGGACTCCACGTTCGCTTGGATCTTATG
TCATGGCCGA............TAGACTGAAGACGT**CTTCTCCTCGTTGCATGCTTT**TGTAGTGTC**AACAAC**
TGGACCGGATATGTTTACAATGGGGAATATCAATAAAAATCTATTTTTCTGAA**GGATCGCGGTT**
CCACATTGTAGATTTGATCTTTAGTAGTTTGGGTAGTTTCTTACGGCAAAAAGTCTAGAATTG
TTTATTCATTTTAAACAAAGGTTTTTACACTTCTATGCAATTGTACAAAAATAGACAGACGGGT
GTGTGATAACATG**TAACATTGCGTGCCATGTCTT**TGTACAGAGTGGAGCGCATTTAAAAGAAAA
AAACTAAAGTCTTTTATCTAAATGGGTGAGTGAGAACACACAGGGGTGCTTTATCAACTCATTT
TGTTTGTAACAAAATAACAAATGCATATTAAAAGGCAA

B.

dmd	Oligos to order	Mature miRNA produced
DMD-RNAi01_F	TGCTGAAAGCATGCAACGAGGAGAAGGTTTTGGCCACTGACTGACCTTCTCCTTTGCATGCTTT	AAAGCATGCAACGAGGAGAAG
DMD-RNAi01_R	CCTGAAAGCATGCAAAGGAGAAGGTCAGTCAGTGGCCAAAACCTTCTCCTCGTTGCATGCTTTC	
DMD-RNAi02_F	TGCTGAACATATCCGGTCCAGTTGTTGTTTTGGCCACTGACTGACAACAACTGCCGGATATGTT	AACATATCCGGTCCAGTTGTT
DMD-RNAi02_R	CCTGAACATATCCGGCAGTTGTTGTCAGTCAGTGGCCAAAACAACAACTGGACCGGATATGTTC	
DMD-RNAi03_F	TGCTGTACAATGTGGAACCGCGATCCGTTTTGGCCACTGACTGACGGATCGCGTCCACATTGTA	TACAATGTGGAACCGCGATCC
DMD-RNAi03_R	CCTGTACAATGTGGACGCGATCCGTCAGTCAGTGGCCAAAACGGATCGCGGTTCCACATTGTAC	
DMD-RNAi04_F	TGCTGAAGACATGGCACGCAATGTTAGTTTTGGCCACTGACTGACTAACATTGTGCCATGTCTT	AAGACATGGCACGCAATGTTA
DMD-RNAi04_R	CCTGAAGACATGGCACAATGTTAGTCAGTCAGTGGCCAAAACTAACATTGCGTGCCATGTCTTC	

Figure S7. Synthetic *pri-miRNAs* design against *dmd*. A. cDNA sequence highlighting miRNA target sites in blues (Coding sequence in Green -truncated here- ; Start codon highlighted in turquoise; Stop codon highlighted in Red; miRNA target sites in Bold blue). **B.** Top and bottom oligo to order for generating the *pri-miRs* to be inserted into BsmBI-digested p3E-IntronMIR / p3E-IntronRNAi.

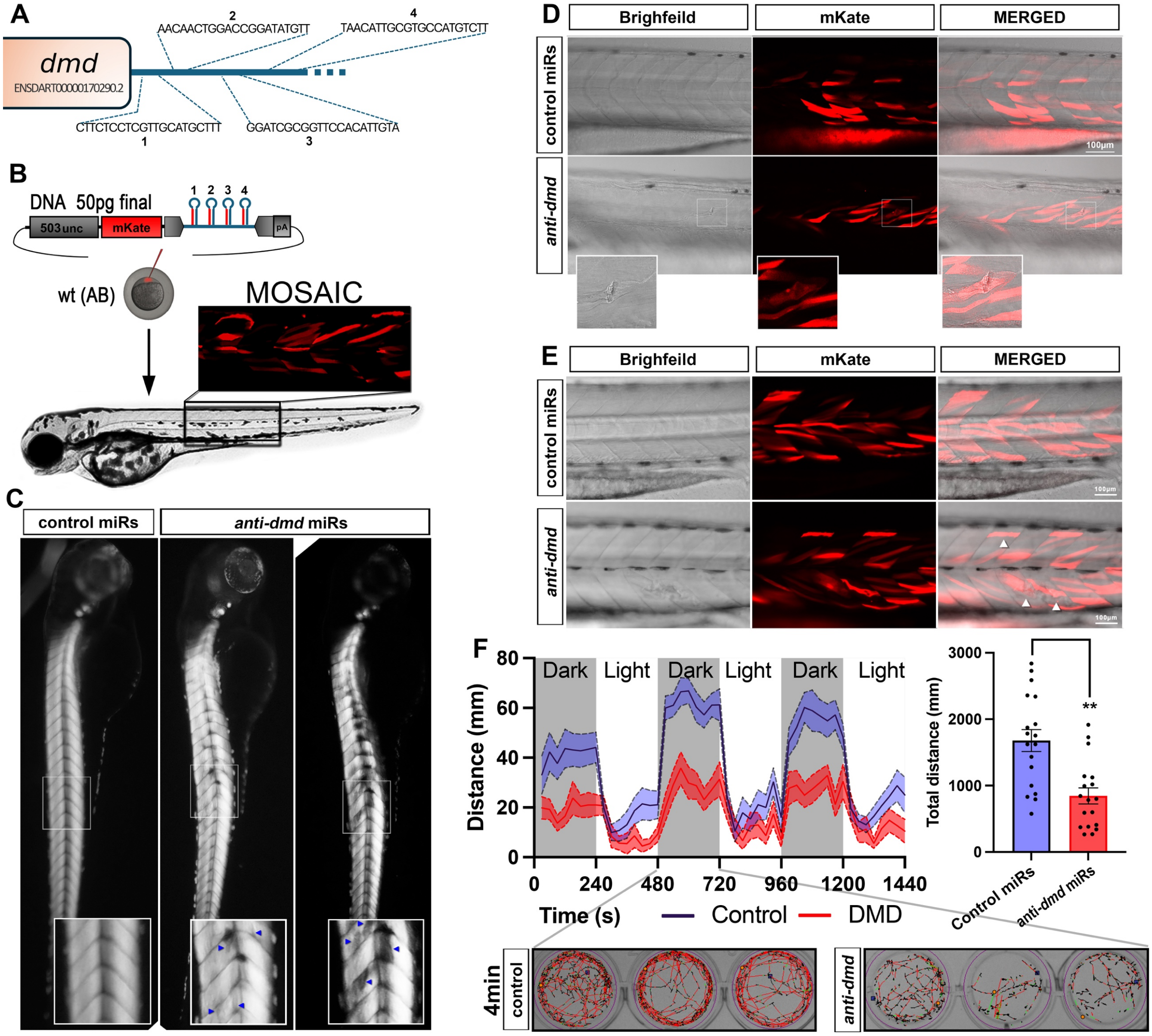

Figure S8. Cell-specific expression of anti-*dmd* miRNAs *triggers muscle fibres detachment similar to stochastic fibres detachment observed in sapje (dmd) mutants*. A. Schematic representation and sequences of the targeted 3'UTR regions of the *dmd* mRNA. **B.** Injection strategy used to trigger mosaic/cell-specific expression of the miR-mediated transgene. **C.** Birefringence snapshots evidencing muscle fibre defects and detachments in 4dpf injected anti-*dmd* animals but not in the controls. **D/E.** Confocal z-stacks/scan evidencing the muscle fibres defects/detachments in the anti-*dmd* group at 3dpf (D) or 4dpf (E). No defects or detachment could be detected in any of the control animals. For those experiments, prior to imaging, animals were anaesthetized with tricaine, embedded into 1% low-melting-agarose (LMT) and mounted in lateral view. **F.** Distance swam by animals identified to have muscle defects (Red lines) versus control (Blue line) n=18. Swimming experiments consisted of 24 minutes records in 24-well plates including 3x repetitive periods of 4min darkness and 4min light conditions.

Video S01. Detaching muscle fibres recorded during time-lapse imaging using an Olympus FV3000 (Brightfield)

Video S02. Detaching muscle fibres recorded during time-lapse imaging using an Olympus FV3000 (mKate)

A.

ENSDART00000124040.3

CGCGCGTCCAAATATAGTACCAGGTGTGCCTCTGATTTCAACCCGACTGAAGTGCCTTTCAGCTGGCGAA
GATGCCCAGAGGATTTTTAGTCAAGCGAAATAAGAAAGCGACACCTGTTTCGTACCGGGTCCGCTCGGAG
GAGGACGAGCAGGGCGCGTTTGTCGCGCAGGATGTTCCGTGCGCGCCGCCCGGTGTCACCCGTGCAGT
TCGGGAACCCCGAGACAGTCTACCGGGCTATGTACAGCCCGACGCGTCCCGTCAGCAGAGAGCACGAGAG
GGCGTGTTTGGAGCGGCGCTTTAATCTCGGCTCGCCCATCTCAGCGGAGTCTTTCCCGGCGGCGCCAAAC
TGCTCCGACCAAGCGCCGGTGGATCTTAAAATCGGCACCAGCAACAGCAACCGAACCGGCACCACAGTAA
CCACCAAAAGACCCGCGTCCGACACAGAGCGCAAAGGCAAACCGGCATCCAAGAAAGCCAAAGCCATGCG
GAAGCTGCAGTTCGAGGATGAGATGACCACCTCTCCAGTGCTCGGACTGAAGATCAAAGAAGGTCCGGTG
GAGCAGAAGCCCAGATCGCAGTGCGCAAGCGGAGACAAGCCGCTCGGAGAGTTCGTCTGTCAGCTGTGCA
GAGAGGCATACGCGGACCCCTTCTCTCTAGCCCAGCACAAATGCTCCAGGATCGTCAGGATCGAGTACAG
ATGTCCCGAATGCGACAAGCTCTTCAGCTGCCCGGCGAACCTCGCCTCGCACCGGCGGTGGCACAAACCC
AAGCAGAGCGCGGAAAGCAATAAAACACCCGCGCCCGAGAAAGAGGAGACTTCCAGCGACAGGGACACTC
CTAGTCCCGGACTTTCCGAAAGCGGCTCTGAGGACGGCCTGTACGACTGCCAGCACTGCGGGAAGAAGTT
CAAGCGTCAGGCGTACCTGAAGAAGCATGTGACTGCGCACCACGACGCGCCAGAAAAACCCCAAAGCCAC
GCGCCTCTGAATCTCAGCGCCTCCGAGTGTCACCTGTGCCCGGTGTGCGGGGAGAACTTCCCGAGCAGGA
TGAGCCAGGAGCGCCACATCCGCCTGCAGCACTCGGCGCAGGTCTACCCGTGCAAATACTGTCCGGCCAT
GTTTTATAGCTCGCCGGGACTTACGAGACACATCAACAAATGCCACCCGTCGGAAAACAGGCAGGTGATC
CTGCTTCAGATGCCGGTGCGTCCAGCCTGCTGAAGACAACCTGTGCCTTAAACCAGTGCTTCCCAGCCCA
CA**ACGTTCCGGATTGGATGGGAT**GGCAGGGGGTCGGGAAGCACTGCCTTAAACCACAGAAT**CAACCTGGA**
TGAGCATCACATGCGAATCCTGCCATTTTCTAATCTACAACTCTCGAAAGGAGTTTTCCGAGTGATGTCA
CAGAAGAATCCGTGTTCGATTCTGGGAAGAACGGCGAACGTTCTAAAACAGAAGTGAGAACGCTTTACGC
AATGGAAAGACTCCAACGCTTGATTTTTTTAAAGAGGGTAAAGTAGTTGATCTGAAGGTGGTACAACAGG
TTAGACTGTTCGCTTCTTTTTCTAGGATCTCCGCACAAGTGCTTTTAGCTTTTAGACCTAAGAATCCTAG
AACATGCCTTTGAATTCACAGGCTGTACATACATTTTTCCATCGTTTCTAGCTTTAATAGAGCTTGTGAA
TCTGTGGACGTTTGACTGTAGCTTTCAAAACAACCTGCTG**ACAAGGGTTTCTCCCTTTAGT**GTAGCCAGA
AATGCCTTGAATTAGATCTCGACATGCTGTAAAACTGCCTTAATCATGTAAATGTCGGA**GTGATTGCGGT**
TACTGATGATGTGTTGTTGTTTTTTTTAATACTATTGTTGTTATTATTATTATTCGCGTCATTGTGTGTG
CGTATATGTTTGTGATTATTTATCTAATTATTATTATTATTATTTATTTATTTCAAATGATTTATTTAAC
TTATTCCTCTGCCTCATTCGTTTGTGATTGATTTTGAAATTTGTCTCGACAGCCAAATGTTTCTTTTATT
GATCTGAAGGTCAATCTCCGAGTCTGAAATCCACCTTGCATTAGGTACAATCCTTTATATTAATTTATTT
TTTTTATGTTACGTCTGACATGCTCAGCGCTTGTGTGTGTTTCTGCAGTTCTGCTATTTGTCCAAAAATT
ACTTATGATAATAATAATAATAACAATAAACAGTCCAAAAAGTCTAATGGGATCCACCTGATGTGACATT
CCAGCAATCTGAACACGTGTGTGTGTTTGTGTGTGTGTGTCGACATGTGGAAACCCAGACACTGTGAACA
CTTACAAATGCTTCATCTTCATCATCTTATCTTCTTCTACTTCTTATACGATGAAATGTTTCCATCTGGA
TTCTGTACCAAGATACGTCTGCACTTAATAAAACACGTCAACTT

B.

insm1a	Oligos to order	Mature miRNA produced
insm1a-01F	TGCTGATCCCATCCAATCCGGAACGTGTTTTGGCCACTGACTGACACGTTCCGTTGGATGGGAT	ATCCCATCCAATCCGGAACGT
insm1a-01R	CCTGATCCCATCCAACGGAACGTGTCAGTCAGTGGCCAAAACACGTTCCGGATTGGATGGGATC	
insm1a-02F	TGCTGATGTGATGCTCATCCAGGTTGGTTTTGGCCACTGACTGACCAACCTGGGAGCATCACAT	ATGTGATGCTCATCCAGGTTG
insm1a-02R	CCTGATGTGATGCTCCCAGGTTGGTCAGTCAGTGGCCAAAACCAACCTGGATGAGCATCACATC	
insm1a-03F	TGCTGAAGCGTTGGAGTCTTTCCATTGTTTTGGCCACTGACTGACAATGGAAACTCCAACGCTT	AAGCGTTGGAGTCTTTCCATT
insm1a-03R	CCTGAAGCGTTGGAGTTTCCATTGTCAGTCAGTGGCCAAAACAATGGAAAGACTCCAACGCTTC	
insm1a-04F	TGCTGTTCAAAGGCATGTTCTAGGATGTTTTGGCCACTGACTGACATCCTAGAATGCCTTTGAA	TTCAAAGGCATGTTCTAGGAT
insm1a-04R	CCTGTTCAAAGGCATTCTAGGATGTCAGTCAGTGGCCAAAACATCCTAGAACATGCCTTTGAAC	
insm1a-05F	TGCTGACTAAAGGGAGAAACCCTTGTGTTTTGGCCACTGACTGACACAAGGGTCTCCCTTTAGT	ACTAAAGGGAGAAACCCTTGT
insm1a-05R	CCTGACTAAAGGGAGACCCTTGTGTCAGTCAGTGGCCAAAACACAAGGGTTTCTCCCTTTAGTC	
insm1a-06F	TGCTGATCATCAGTAACCGCAATCACGTTTTGGCCACTGACTGACGTGATTGCTTACTGATGAT	ATCATCAGTAACCGCAATCAC
insm1a-06R	CCTGATCATCAGTAAGCAATCACGTCAGTCAGTGGCCAAAACGTGATTGCGGTTACTGATGATC	

Figure S9. Synthetic *pri-miRNAs* design against *insm1a*. A. cDNA sequence highlighting miRNA target sites in blues (Coding sequence in Green -truncated- ; Start codon highlighted in turquoise; Stop codon highlighted in Red; miRNA target sites in Bold blue). **B.** Top and bottom oligo to order for generating *pri-miR* to be inserted into BsmBI-digested p3E-IntronMIR / p3E-IntronRNAi (6 repeats).

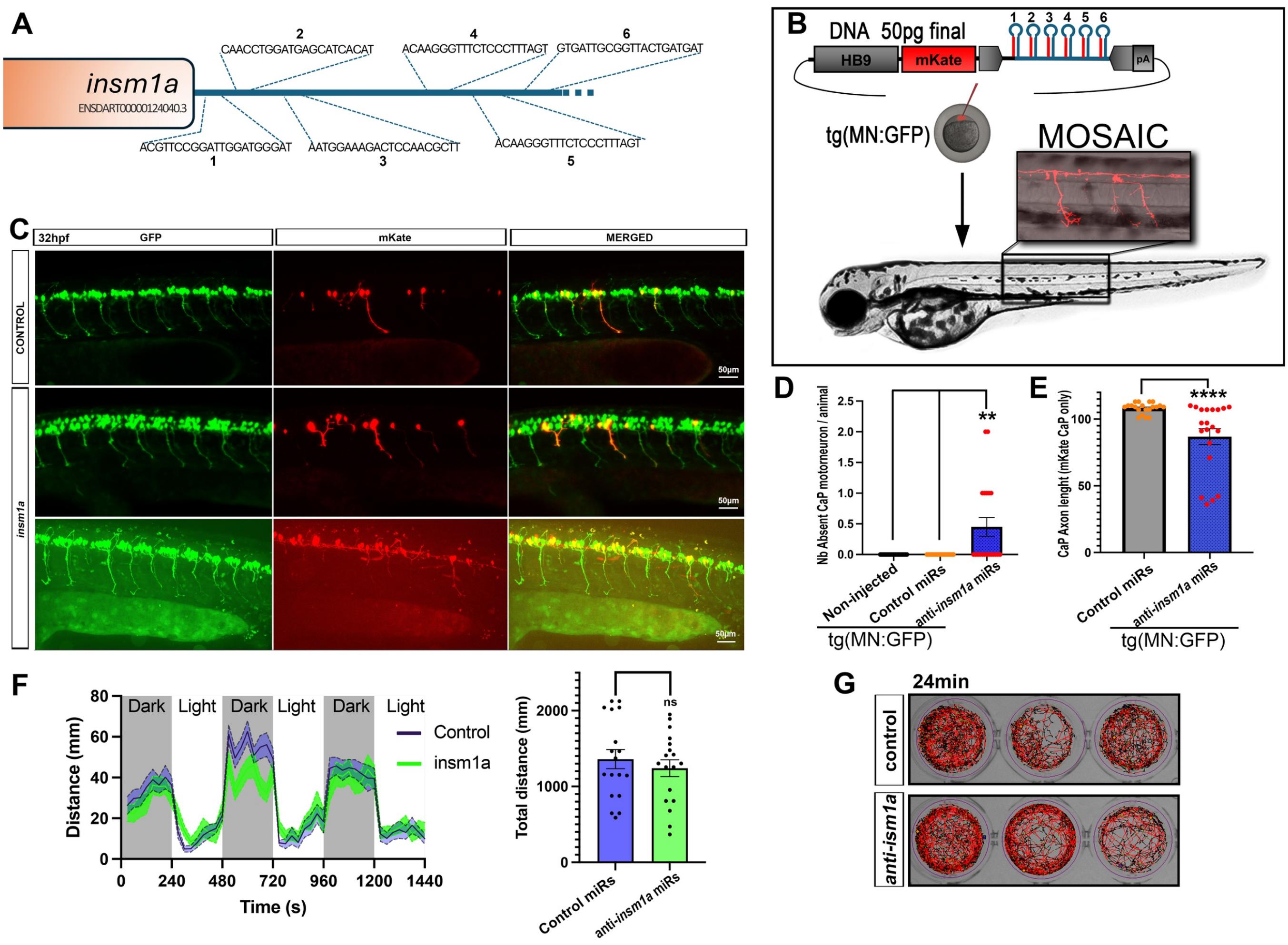

Figure S10. Cell-specific expression of anti-*insm1a* miRNAs impact motor neuron development. A. Schematic representation and sequences of the targeted 3'UTR regions of the *insm1a* mRNA. **B.** Injection strategy used to trigger mosaic/cell-specific expression of the miR-mediated transgene. **C.** Confocal Max-Intensity projections (Latera views, 32hpf) evidencing CaP motor neuron defects observed in injected anti-*insm1a* animals. **D.** Number of absent CaP Motor neurons observed in non-injected versus injected control and anti-*insm1a* animals. **E.** CaP axon length (µm, average) measurement of mKate positive CaP neurons in control versus anti-*insm1a* animals. Non-injected animals do not present any mKate-positive neurons. **F-G.** Distance swam by mKate positive controls vs anti-*insm1a* animals (Blue line) n=18. Swimming experiments consisted of 24 minutes records in 24-well plates including 3x repetitive periods of 4min darkness and 4min light conditions.
